# Fibromyalgia: Pathogenesis, Mechanisms, Diagnosis and Treatment Options Update

**DOI:** 10.3390/ijms22083891

**Published:** 2021-04-09

**Authors:** Rosalba Siracusa, Rosanna Di Paola, Salvatore Cuzzocrea, Daniela Impellizzeri

**Affiliations:** 1 Department of Chemical, Biological, Pharmaceutical and Environmental Sciences, University of Messina, Viale Ferdinando Stagno D’Alcontres, 31, 98166 Messina, Italy; rsiracusa@unime.it (R.S.); dimpellizzeri@unime.it (D.I.); 2 Department of Pharmacological and Physiological Science, Saint Louis University School of Medicine, Saint Louis, MO 63104, USA

**Keywords:** central sensitization, cognitive-emotional sensitization, biomarkers, genetic aspects, therapy

## Abstract

Fibromyalgia is a syndrome characterized by chronic and widespread musculoskeletal pain, often accompanied by other symptoms, such as fatigue, intestinal disorders and alterations in sleep and mood. It is estimated that two to eight percent of the world population is affected by fibromyalgia. From a medical point of view, this pathology still presents inexplicable aspects. It is known that fibromyalgia is caused by a central sensitization phenomenon characterized by the dysfunction of neuro-circuits, which involves the perception, transmission and processing of afferent nociceptive stimuli, with the prevalent manifestation of pain at the level of the locomotor system. In recent years, the pathogenesis of fibromyalgia has also been linked to other factors, such as inflammatory, immune, endocrine, genetic and psychosocial factors. A rheumatologist typically makes a diagnosis of fibromyalgia when the patient describes a history of pain spreading in all quadrants of the body for at least three months and when pain is caused by digital pressure in at least 11 out of 18 allogenic points, called tender points. Fibromyalgia does not involve organic damage, and several diagnostic approaches have been developed in recent years, including the analysis of genetic, epigenetic and serological biomarkers. Symptoms often begin after physical or emotional trauma, but in many cases, there appears to be no obvious trigger. Women are more prone to developing the disease than men. Unfortunately, the conventional medical therapies that target this pathology produce limited benefits. They remain largely pharmacological in nature and tend to treat the symptomatic aspects of various disorders reported by the patient. The statistics, however, highlight the fact that 90% of people with fibromyalgia also turn to complementary medicine to manage their symptoms.

## 1. Fibromyalgia

**Figure 1:**
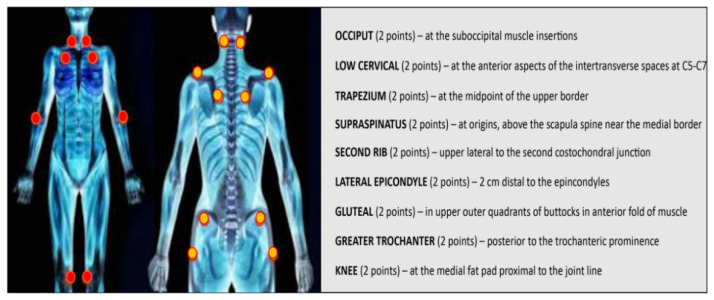
The dots indicate the 18 tenderness points important for the diagnosis of FM.

Fibromyalgia (FM) is a syndrome characterized by chronic musculoskeletal pain. The main symptoms of this disease are muscle stiffness, joint stiffness, insomnia, fatigue, mood disorders, cognitive dysfunction, anxiety, depression, general sensitivity and the inability to carry out normal daily activities [1,2]. FM can also be associated with specific diseases, such as infections, diabetes, rheumatic diseases and psychiatric or neurological disorders [3]. FM was first described in the 19th century. In the 1970s and 1980s, an etiology of the disease involving the central nervous system was discovered [4]. In 1950, Graham introduced the concept of “pain syndrome” in the absence of a specific organic disease [5]. The term “fibromyalgia” was later coined by Smythe and Moldofsky following the identification of regions of extreme tenderness known as “pain points” [6]. These points are defined as areas of hyperalgesia/allodynia when a pressure of about 4 kg causes pain [7]. In 1990, the committee of the American College of Rheumatology (ACR) drew up diagnostic criteria, which have only recently been modified [8,9]. According to the ACR, the diagnosis of FM includes two variables: (1) bilateral pain above and below the waist, characterized by centralized pain, and (2) chronic generalized pain that lasts for at least three months, characterized by pain on palpation in at least 11 of 18 specific body sites [10] (Figure 1). FM affects about 5% of the world population. The incidence is higher in women than in men, and the age range in which FM generally appears is between 30 and 35 years [11]. However, FM remains a poorly understood and difficult-to-diagnose condition.

## 2. Pathophysiology

The pathophysiological factors of FM are not yet well known and continue to be the focus of much research. FM appears to be related to a pain-processing problem in the brain. In most cases, patients become hypersensitive to pain. The constant hypervigilance to pain can also be associated with psychological problems [12].

The main alterations observed in FM are dysfunctions in mono-aminergic neurotransmission, leading to elevated levels of excitatory neurotransmitters, such as glutamate and substance P, and decreased levels of serotonin and norepinephrine in the spinal cord at the level of descending anti-nociceptive pathways. Other anomalies observed are dopamine dysregulation and altered activity of endogenous cerebral opioids. Taken together, these phenomena seem to explain the central physiopathology of FM [13].

Over the years, peripheral pain generators have also been recognized as a possible cause of FM. In this case, patients manifest symptoms such as cognitive impairment, chronic fatigue, sleep disturbances, intestinal irritability, interstitial cystitis and mood disorders [14,15].

Peripheral abnormalities may contribute to increased nociceptive tonic supply in the spinal cord, which results in central sensitization. Other factors that appear to be involved in the pathophysiology of FM are neuroendocrine factors, genetic predisposition, oxidative stress and environmental and psychosocial changes [16,17].

FM appears to be more common in women than men for the following reasons: higher levels of anxiety and depression, altered behavior in response to pain, altered CNS input and hormonal effects related to the menstrual cycle [12].

### 2.1. Principal Processes Underlying FM

**Figure 2:**
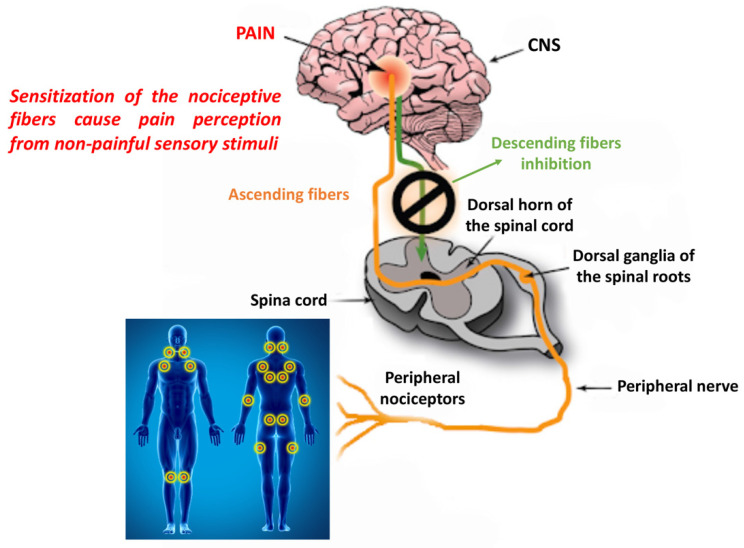
Ascending and descending pathways that influence pain sensitivity.

As mentioned above, FM is considered a central sensitivity syndrome. Central sensitization refers to a neuronal signal amplification mechanism within the central nervous system that leads to a greater perception of pain [18]. For this reason, patients with FM present an increase in the receptive field of pain, allodynia and hyperalgesia. Central sensitization is also implicated in persistent and chronic pain. Although central sensitization plays an important role in FM, it is even more important to understand the initial cause, that is, the persistent nociceptive input associated with tissue damage, including peripheral sensitization [19] (Figure 2). According to Vierck, if peripheral pain generators can be blocked, the symptoms of FM should disappear or not even develop. Despite this, researchers focus more on central sensitization as the mechanism of pain sensitivity because there is less evidence to support the involvement of peripheral pain tissue abnormalities and nociceptive processes in FM [20]. However, sensitization is not a unitary phenomenon, and a distinction must be made between central, peripheral and psychosocial sensitization [21,22]. Brosschot et al. observed that FM patients were selectively attentive to information regarding the body and the environment in relation to pain. In this regard, they introduced the term “cognitive-emotional sensitization” to explain how selective attention to certain body pain can increase the perception of that pain. Pain sensitivity is also linked to social groups. It has been suggested that the mechanism that underlies “interpersonal sensitization” could be linked to the shared neuronal representation of the experience of pain. In other words, a feed-forward effect occurs in which a family, in an attempt to reduce painful behaviors in one of its members, actually creates a state of anxiety in the person concerned by increasing the perception of pain [23,24].

#### 2.1.1. Peripheral and Central Sensitization

Patients with FM present a lower pain threshold that generates a condition of diffuse hyperalgesia and/or allodynia. This indicates that there may be a problem with the amplification of pain or with sensory processing in the CNS. These FM phenomena have been confirmed in clinical studies that used functional neuroimaging or measured alterations in neurotransmitter levels that influence sensory transmission and pain [25,26,27]. It was also observed that treatments with drugs aimed at increasing anti-nociceptive neurotransmitters in the CNS or at lowering the levels of pro-nociceptive excitatory neurotransmitters, such as glutamate, were able to improve these conditions in patients with FM. Exercise has also proved useful for increasing anti-nociceptive neurotransmitters and reducing glutamate [28,29]. In contrast, patients with FM do not respond to non-steroidal anti-inflammatory drug (NSAID) therapies aimed at resolving acute pain or pain induced by tissue damage or inflammation. Animal models of non-inflammatory pain, such as that induced by repeated injections of acidic saline (pH 4 or pH 5) into the gastrocnemius muscle, have been used to mimic the clinical signs and symptoms observed in FM [30,31,32,33]. These studies reported that widespread and lasting hyperalgesia was greater in female mice than in males [31,32]. In addition, a pharmacological response similar to the clinical one was observed in animal models of non-inflammatory pain used for FM. In particular, a reduction in pain and hyperalgesia was observed following treatment with antidepressants, opioids, glutamate receptor antagonists and Na^+^ channels, while no effect was observed with NSAIDs [34,35,36].

Clinical studies based on functional magnetic resonance imaging (fMRI) have confirmed a central neuronal alteration in nociceptive processes. In particular, following the same amount of pressure stimuli, patients with FM had greater neuronal activation in the pain-processing areas of the brain than control subjects [37,38]. In individuals with diffuse hyperalgesia or allodynia, the main brain regions in which greater neuronal activity is observed are the posterior insula and secondary somatosensory cortices [39,40]. The subtle differences in fMRI results in studies on FM or other chronic pain conditions are due to the fact that the stimulus to induce pain is not normalized and therefore may differ in intensity with each scan. fMRI studies have also been useful for determining the involvement of psychological factors in pain processing in FM [40,41,42,43].

The use of fMRI has also been useful for examining the degree of connection between the various brain regions. This analysis has been applied to both resting and active individuals. The advantage of the analysis at rest is that it provides data on brain changes associated with ongoing spontaneous chronic pain. With this technique, individuals with FM were shown to have greater connectivity between a network called “default mode” (active when the brain is at rest) and the insula (pro-nociceptive region) [44]. The degree of connectivity between these regions depends on the intensity of spontaneous and continuous pain [45]. It was later found that other brain regions may be hypo-connected in individuals with FM. For example, following a painful stimulus, a reduction in connectivity is observed between the anti-nociceptive areas in the brain stem in patients with FM. This suggests that there is a defect in the descending inhibitory systems in this condition [46]. Additionally, in clinical studies, an increase in glutamate levels has been observed in the brains of FM patients [47,48,49]. Using proton magnetic resonance spectroscopy, these levels were observed to increase in the main areas of pain processing, such as the insula [50,51,52]. 

Furthermore, pregabalin has been observed to reduce glutamatergic activity in the insula and functional connectivity between the default mode network and the insula in patients with FM [53]. Another important clinical observation is that some subgroups of patients with FM respond to treatment with N-methyl-D-aspartate (NMDA) glutamate receptor antagonists, suggesting an increase in glutamatergic activity. However, the use of these drugs is not always well tolerated and therefore may not always have a clinical use [54,55,56]. Alternatively, a low-glutamate diet has been shown to reduce FM symptoms [57]. Parallel to the clinical evidence, animal studies in the non-inflammatory pain model, which is used to induce the typical symptoms of FM, have shown increased glutamate release in the spinal and ventromedial rostral cords [58,59]. In particular, in non-inflammatory pain models that involve two injections (administered five days apart) of acidic saline into the gastrocnemius muscle, an increase in glutamate levels was observed after the second injection of acidic saline but not after the first. This increase corresponds to the development of prolonged diffuse hyperalgesia that occurs after the second injection. This suggests that greater excitability in the CNS induced by a single low-intensity muscle insult causes the same nervous system to mount an exaggerated response to a subsequent occurrence of the same stimulus. As in FM patients, experimental animals respond to the blockade of NMDA receptors [60,61,62]. An important role in the excitability of neurons is played by the NR1 subunit of the NMDA receptor, which is necessary for the formation of the receptor itself and its insertion into the synapse. In an animal study, recombinant lentiviruses were used to overexpress the NR1 subunit, and the nociceptive sensitivity to skin and muscle stimuli was measured to demonstrate that the increased expression of the NR1 subunit in the ventromedial rostral medulla is critical for the development of hypersensitivity. The results showed that NR1 overexpression in the rostral ventromedial medulla reduced the muscle and skin withdrawal threshold, leading to diffuse hyperalgesia similar to that observed in the non-inflammatory pain model. Furthermore, in the same study, the expression of NR1 was downregulated, and the hyperalgesia induced by repeated muscle injections of acid saline (pH 4.0) was measured. The results showed that downregulation of this subunit increased the muscle withdrawal threshold, preventing the onset of hyperalgesia [61,62]. Taken together, these data provide evidence that NMDA glutamate receptors in the CNS play a central role in the induction and maintenance of widespread hyperalgesia. Another fundamental role that has emerged from in vivo studies is that played by intracellular messengers, whose changes can produce lasting effects by altering neuronal excitability and improving gene transcription. In an FM animal model induced by restraint stress with intermittent cold stress, brain-derived neurotrophic factor (BDNF) and phospho-cAMP response element-binding protein (p-CREB) proteins were downregulated in the medial prefrontal cortex and hippocampus of animals with FM compared to the control group. Therefore, this animal model of FM could be used to investigate the BDNF–CREB pathway and pain [63]. Studies of acute paw inflammation induced by carrageenan [64] or subcutaneous formalin [65,66] and studies on neuropathic pain [67,68] have shown an increase in phosphorylated CREB due to the activation of the cAMP pathway. On the basis of these studies, a group of researchers aimed to evaluate the role of the cAMP pathway in mechanical hyperalgesia and in the phosphorylation of the transcription factor CREB in a model of chronic muscle pain (two injections of sterile saline at pH 4.0) which mimics the typical chronic muscle pain of patients with FM. The results of this study demonstrate that CREB phosphorylation is time-dependent and occurs in parallel to the cAMP-dependent phase of mechanical hyperalgesia. The increase in CREB phosphorylation is reversed by blocking the cAMP pathway and correlates with the mechanical withdrawal threshold. Therefore, the increase in phosphorylated CREB may contribute to mechanical hyperalgesia. These data have some clinical relevance, as they suggest that modulation of the cAMP pathway may be useful in the early stages of muscle hyperalgesia [69]. In addition, an increase in the phosphorylation of ERK, an intracellular signaling molecule, was also observed at the level of the paraventricular thalamus and in the amygdala [70,71]. This phenomenon and hyperalgesia can be prevented by the intracerebroventricular blockade of T-type Ca^2+^ channels [70]. This latter observation suggests that Ca^2+^ channels may mediate some of these changes. Therefore, the animal models studied show that alterations in central excitability occur throughout the nociceptive system, from the spinal cord to the cortex.

Although FM is thought to be a central pain disorder, many studies have shown peripheral nerve changes in patients with FM. In particular, people with FM have been reported to have a reduced number of epidermal nerve fibers in skin biopsies [72,73,74,75]. Clinical observations have also shown that FM patients score higher on neuropathic pain questionnaires and have altered heat, cold and pain thresholds [72,73]. By using microneurography on patients, some researchers have shown that mechanically insensitive C-fibers have higher spontaneous activity and increased sensitization to mechanical stimulation [76]. The notion that peripheral factors may underlie pain in FM is supported by the observation that the administration of lidocaine into the muscles of patients with FM significantly reduced hyperalgesia at the local site, and moreover, the pain perceived outside the injection site was reduced by 38% [77]. In a study investigating changes in muscle tissue, no differences were observed in the number of type I or II fibers or in the capillary density between individuals with FM and healthy subjects [78]. However, other clinical studies have shown that resistance to fatigue was closely related to the size of type I muscle fibers and the oxygenation of hemoglobin. FM patients who had a higher percentage of type I fibers were shown to recover strength more efficiently. According to these researchers, these measures may relate to the fatigue typical of FM. Although no differences were noted in measurements of performance or muscle fatigue between subjects with and without FM, individuals with FM, especially women, reported increased fatigue and pain during exercise [79,80]. Self-reported fatigue is generally thought to be caused by the CNS since these symptoms often respond to centrally acting drugs, but according to the latest data, these symptoms are possibly due to changes in muscle tissue.

In humans, the intramuscular injection of an acid solution into the anterior tibial muscle induces muscle pain both at the site and in the ankle, producing primary and secondary hyperalgesia [81]. Therefore, a decrease in pH can contribute to hyperalgesia and referred pain. It has been reported that some acid-sensing ion channel (ASIC) members are activated following very slight acidification (from pH 7.4 to 7.2) and generate a depolarizing current at the peripheral terminals of nociceptors, leading to the detection of pain [82,83,84]. A greater number of acid-sensing ion channel 3 (ASIC3) receptors have been observed on sensory neurons innervating the skeletal muscle than on those innervating the skin. Furthermore, most of these afferents that innervate skeletal muscle and express ASIC3 also co-express calcitonin gene-related peptide (CGRP), a nociceptive marker [85,86]. The important role of ASIC3 activation on nociceptors that innervate the skeletal muscle in the induction of diffuse hyperalgesia was suggested by the results of an animal study, which demonstrated that blocking ASIC3 with APETx2 was capable of inhibiting the development of hyperalgesia [87,88,89]. Furthermore, the importance of ASIC3 activation was also observed in the repeated acid model or fatigue-induced model, as hyperalgesia was not detected in ASIC3 knockout mice [89,90].

However, it has been shown that an ASIC antagonist administered after the onset of hyperalgesia has no positive effect. This indicates that once hyperalgesia has developed, it is independent of the activation of nociceptors due to acid pH [87,91]. In contrast, in a reserpine-induced FM model, ASIC3 mRNA was highly expressed in the spinal dorsal root ganglion (DRG), and hyperalgesia was reversed by administering APETx2, thereby blocking ASICs. Furthermore, the mechanical reactivity of C fibers in this model was a greater in both the skin and the muscles [92]. These results therefore show that the ASIC3 present at the peripheral level could modulate widespread hyperalgesia. Indeed, the increased reactivity of C fibers at the skin level is similar to that observed in subjects with FM, and this suggests that ASIC3 may be involved in modulating altered peripheral sensitivity.

The release of substance P from nociceptive nerve fibers and the activation of its neurokinin 1 receptor (NK1), which is found post-synaptically in the spinal dorsal horn, as well as on immune cells, smooth muscle, blood vessels and other peripheral cells, seem to play an important role in the transmission of pain signals [93]. Substances that inhibit substance P signaling pathways usually produce anti-nociceptive effects in animal models [94,95]. Therefore, substance P is believed to promote pain. On the other hand, NK1 antagonists have failed to produce analgesic effects in many clinical studies; therefore, its function has yet to be clarified [96]. An unexpected anti-nociceptive role of substance P was observed in muscle-afferent DRG neurons. In a mouse model of chronic mechanical hyperalgesia induced by repeated intramuscular acid injections, the release of substance P in the muscle appeared to play a physiological role in nociceptive plasticity and limited the activation of muscle nociceptors induced by acid injection. This physiological mechanism could be of therapeutic interest, as the administration of a selective NK1 agonist prevents long-lasting hyperalgesia. In support of this hypothesis, animal studies have shown that a single intramuscular injection of acid in tachykinin precursor 1 gene (Tac1) knockout mice, which therefore lack substance P signaling, or the co-administration of NK1 receptor antagonists induces long-lasting rather than transient hyperalgesia, as occurs in controls. The anti-hyperalgesic effect of substance P was observed only in neurons expressing ASIC3, where substance P increases M-channel-like potassium currents through the NK1 receptor independently of protein G but in a tyrosine kinase-dependent manner. Thus, through these works, intramuscular substance P was confirmed to mediate an unconventional NK1 receptor signaling pathway that led to an unexpected anti-nociceptive effect against chronic mechanical hyperalgesia induced by repeated acid injections [97]. 

As previously mentioned, the chronic pain typical of FM is due to alterations in central and peripheral sensitization. Over the years, researchers have searched for biomarkers that are capable of detecting these changes. In particular, they focused on factors capable of acting on the growth and survival of nerve cells, such as nerve growth factor (NGF). This factor is indeed involved in promoting the growth, proliferation and survival of sensory neurons that transmit pain, temperature and tactile sensations [98].

Data obtained in earlier studies showed an increase in NGF in the cerebrospinal fluid of FM patients [99]. However, these data disagree with those from a recent study in patients, in which plasma NGF levels were not found to differ between FM and control subjects. In this regard, different statistical methods were used, which nonetheless led to the same conclusions [100]. Further studies are therefore needed to understand the involvement of NGF in the pathophysiology of FM.

#### 2.1.2. Inflammation and Immunity 

Increasing evidence indicates that neurogenic-derived inflammatory processes occurring in the peripheral tissues, spinal cord and brain are also responsible for the pathophysiology of FM [101,102,103]. In fact, the release of biologically active agents, such as chemokines and cytokines, leads to the activation of the innate and adaptive immune system. All of this translates into many of the peripheral clinical features reported by patients with FM, such as swelling and dysesthesia, which can also affect central symptoms, including cognitive changes and fatigue. In addition, the physiological mechanisms related to stress and emotions are considered to be upstream drivers of neurogenic inflammation in FM [104].

Studies conducted in patients have confirmed that inflammation is involved in FM. Indeed, FM patients have been shown to have enhanced circulating inflammatory cytokines and inflammatory cytokines released by circulating immune cells [103,105].

Kadetoff et al. described an increased concentration of IL-8 in the cerebrospinal fluid of FM patients compared to healthy subjects [106]. This finding could be due to the activation of glial cells, which play an important role in the central sensitization process, as they are activated in response to excitatory synaptic signals (glutamate) [107]. Furthermore, since the synthesis of IL-8 is dependent on orthosympathetic activation, this could help explain the correlation between stress and fibromyalgia symptoms [108]. In addition to this, some studies have shown an increase in serum concentrations of IL-6, IL-8, IL-1β and TNF-α in individuals with FM, although no clear correlation with symptom severity has been identified, except, perhaps, for IL-6 [103,109,110,111]. It appears that immune cells such as mast cells, monocytes and neutrophils, as mediators of inflammation processes, may also have a function in defining an inflammatory substrate of fibromyalgia [112]. In animals, resident macrophages located in the muscle have been shown to contribute to the development of chronic widespread muscle pain. For example, the removal of macrophages at the acid injection site through a local injection of clodronate liposomes is capable of preventing the development of exercise-induced hyperalgesia [89]. Another observation is that pro-inflammatory cytokines, such as interleukins (IL-1β, IL-6) and tumor necrosis factor (TNFα), can activate and sensitize nociceptors, induce pain in humans and trigger hyperalgesia in animals. Another potential source of these cytokines is adipose tissue; many studies suggest that diffuse or multifocal pain is more common in obese individuals [113], and obese animals show enhanced nociceptive responses [114,115]. Therefore, pro-inflammatory cytokines could play a role in the generation of chronic muscle pain, including FM. 

Finally, a study by Smart et al. described a subgroup of fibromyalgia patients characterized by ANA (anti-nuclear antibody) positivity, with the speckled pattern clearly predominating. The use of the Smart Index, which corrects the erythrocyte sedimentation rate value in relation to age, revealed that ANA-positive FM patients had a more pronounced inflammatory response profile than the ANA-negative subgroup, suggesting that autoimmunity potentially contributes to sub-inflammatory fibromyalgia [116]. 

#### 2.1.3. Genetic Aspects 

Over the years, studies have shown the potential involvement of genetic factors in the onset of FM [117,118]. Linkage studies have shown a correlation rate of 50% between genetic variants and the development of chronic pain [119]. Currently, about 100 genes that regulate pain are believed to be relevant to pain sensitivity or analgesia. The main genes are those encoding for voltage-dependent sodium channels, GABAergic pathway proteins, mu-opioid receptors, catechol-O-methyltransferase and GTP cyclohydrolase 1 [120]. The small sample sizes did not allow the authors to confirm an association between single nucleotide polymorphisms and FM susceptibility. However, a genome-wide linkage scan study found that first-degree relatives had an increased risk of developing FM, reinforcing the genetic hypothesis. The serotonin transporter gene (*SLC64A4*) and the transient receptor 2 potential vanillic channel gene (*TRPV2*) are the major genes responsible for pain susceptibility in FM [121]. *SLC64A4* is characterized by a single nucleotide polymorphism and is associated with chronic pain conditions (for example, mandibular joint disorder), as well as increased levels of depression and psychological disorders related to an alteration in serotonin reuptake [122]. The *TRPV2* gene is expressed in mechano- and thermo-responsive neurons in the dorsal root and trigeminal ganglia and appears to be responsible for reducing the pain threshold in FM patients [123]. Other genetic polymorphisms that have been identified and associated with FM susceptibility are in the serotonin transporter (5-HTT), catechol-O-methyltransferase (COMT) and serotonin 2A (5-HT2A) genes. However, subsequent meta-analyses could only confirm that the 102T/C polymorphism in the 5-HT2A receptor is connected with FM [124]. Therefore, further studies are needed to understand the role of these genes in chronic pain conditions such as FM. A genome-wide association and copy number variant study in 952 FM cases and 644 controls revealed the existence of two variables associated with FM. One variable is the single nucleotide polymorphism *rs11127292* in a gene similar to myelin transcription factor 1 (MYT1L), which is responsible for neuronal differentiation and involved in cognitive alterations. The second is an intron copy number variable in the neurexin 3 (*NRXN3*) gene, which normally acts as a receptor and cell adhesion molecule in the nervous system, and variations in this gene are involved in autism spectrum disorder [125]. Other researchers analyzed 350 other genes that are specifically involved in pain treatment. Among these is the *TAAR1* gene, which mediates the availability of dopamine, whose reduction can increase the sensitivity to pain typical of FM [126]. Another widely studied gene is *RGS4*, which is expressed in the dorsal horn of the spinal cord, the locus coeruleus and the nuclei of the bed of the stria terminalis, and is responsible for modulating the descending inhibition of pain perception [127]. One gene studied and related to pain disorders is *CNR1*, which encodes the cannabinoid receptor CB-1 [128,129]. Another gene presumably involved in central sensitization is GRIA4, which mediates the rapid excitatory transmission of nociceptive signals in the central nervous system [130]. Taken together, these studies have increased the current knowledge on FM and support the genetic hypothesis as a potential factor in the pathogenesis of this disease. However, as FM remains a multifactorial disease, further studies are needed to examine haplotypes and combinations of different variants that could influence its development.

#### 2.1.4. Endocrine Factors 

The role of stress in the exacerbation of fibromyalgia symptoms has been widely described from an epidemiological point of view through both self-reports and clinical questionnaires. On the basis of these data, the hypothalamic–pituitary–adrenal axis, central to the stress response, was examined. Despite the discrepancy between different studies on possible alterations in plasma cortisol levels in FM patients, dysregulation of its circadian variation is frequently observed. In particular, flattening of the plasma cortisol concentration curve was observed during the day: this seems to manifest itself through a milder and more gradual descent compared to the morning peak of maximum concentration or through a lowering of the peak itself [131,132,133,134,135]. In addition to this, decreased cortisol secretion has also been described in response to adrenocorticotropic hormone (ACTH) tests [136]. The hypothalamic–pituitary–adrenal axis (HPA) comprises neurotransmitter and neuroendocrine response systems to stress and can be activated in FM [137]. This system may explain some of the symptoms seen in FM.

A patient study looked at levels of corticotropin-releasing factor (CRF) in cerebrospinal fluid (CSF), heart rate variability (HRV) and pain symptoms (e.g., fatigue and depression) in subjects with FM. The results obtained in this study showed that CRF levels were associated with sensory and affective pain symptoms but not with symptoms of fatigue. Furthermore, an increase in HRV was associated with an increase in CRF and pain in patients with FM. These results were subsequently adjusted for age, sex and depressive symptoms, and a correlation between CRF levels and sensory pain symptoms was confirmed. Another important finding was that women with FM and self-reported histories of physical or sexual abuse did not have increased levels of CRF in their CSF. This indicates that there may be subgroups of FM patients with different neurobiological characteristics. Therefore, further studies are needed to better understand the association between CRF and pain symptoms in FM [138]. In another study, the association between salivary cortisol levels and pain symptoms in patients with FM was assessed at different times of day. The results obtained in this study revealed a strong relationship between salivary cortisol and pain symptoms only at the time of awakening and the 1 h that followed in women with FM. Furthermore, no relationship was observed between the cortisol level and symptoms of fatigue or stress. These findings suggest that early-day pain symptoms are associated with changes in HPA function in women with FM.

However, to date, the results regarding the involvement of the HPA in the pathophysiology of FM have been conflicting, and new studies will be needed in the future to fully clarify this aspect [133]. Furthermore, there are indications that total and free cortisol levels are dissociated in FM patients. They have normal salivary and free plasma cortisol despite having reduced total cortisol levels. A possible explanation for this finding is a reduced concentration of glucocorticoid-binding globulin (CBG). Reduced levels of CBG have been reported in FM patients compared to healthy patients. It is of particular interest that chronic social stress can lead to reduced levels of CBG, while IL-6 and IL-1β, which can also inhibit CBG production, may contribute further [139].

The possible pathogenetic role of the growth hormone (GH)/insulin-like growth factor 1 (IGF-1) axis was also investigated. Several studies have found that about one-third of individuals with fibromyalgia have lower IGF-1 levels than control groups [140]. Serial measurements at 12 to 24 h also showed a reduction in GH secretion in patients with fibromyalgia, particularly at night. Since GH secretion occurs mainly during phase 3 of sleep and 80% of patients have sleep disturbances, it remains to be clarified whether the nature of this alteration is primary or secondary [141]. Given the higher prevalence of fibromyalgia in the female population, the role of estrogens in this pathology was investigated. However, the results of various studies suggest that this role is limited, and the only significant result is an increased serum concentration of the G protein-coupled estrogen receptor (GPER) in patients with fibromyalgia compared to healthy subjects [142]. Although a strong correlation with the disease allows us to hypothesize the possible use of this receptor as a potential diagnostic biomarker, the exact mechanism by which it fits into the pathophysiological cascade remains unclear [143].

#### 2.1.5. Psychopathological Factors and Poor Sleep

As previously highlighted, psychiatric comorbidities in fibromyalgia constitute a relevant aspect of the disease, and a close correlation between stress and fibromyalgia symptoms has been described several times. According to several studies, the prevalence of psychiatric comorbidities, such as anxiety disorders and depression, among patients with this pathology reaches 60% in certain subpopulations [144]. The presence of depressive patterns has been shown to correlate with a worse prognosis: patients with comorbid symptoms of depression seem to report pain of greater severity and duration as well as a greater degree of hyperalgesia/allodynia than healthy controls. Furthermore, these psychiatric aspects seem to have a certain predictive value in relation to various somatic symptoms, including musculoskeletal pain and headaches [145]. The impact of depression symptoms on pain processing is still unclear. A study in FM patients attempted to evaluate this correlation by comparing the results of quantitative sensory tests and neuronal responses to pressure stimuli (assessed by functional magnetic resonance imaging (fMRI)) with the levels of symptoms of depression. The results showed that the symptom levels of depression were not associated with quantitative test results or with the extent of neuronal activation in brain areas, such as primary and secondary somatosensory cortices, that are associated with the sensory dimension of pain. However, symptoms of depression were observed to be associated with the extent of pain-evoked neuronal activation in the amygdala and contralateral anterior insula, which are brain areas associated with affective pain processing. Therefore, these findings suggest the existence of parallel, possibly independent, neuronal pain processing networks for sensory and affective pain elements [146]. Finally, the therapeutic aspect is an element that supports the pathogenetic overlap between depressive disorder and fibromyalgia. The efficacy of treatment with antidepressant drugs (e.g., serotonin-norepinephrine reuptake inhibitors (SNRIs) and tricyclics) has in fact been described by numerous studies on FM patients and constitutes one of the main therapeutic strategies in both FM and other chronic pain conditions, such as chronic headache and irritable bowel (IBD), which are often symptoms of FM [147,148,149]. The efficacy of SNRIs and other double-acting antidepressants, such as mirtazapine, suggests that neurotransmission dysfunction of both serotonin and norepinephrine exists in FM [150]. Similar to observations for the depressive pattern, stress also appears to be both a predictive and negative prognostic factor. It has been shown that stress can modulate pain sensitivity by inducing hyperalgesia or allodynia through alterations in the physiological circadian secretion of cholesterol, therefore indirectly inducing the release of pro-inflammatory cytokines and setting in motion the pathophysiological processes described above [151,152,153]. Animal studies have shown that stress induction (e.g., swimming stress and cold stress) can produce muscle and skin hyperalgesia that lasts for weeks after the stressor [154,155,156]. On the other hand, milder stressors (e.g., fatigue and acoustic stress), which do not produce hyperalgesia on their own, can cause an increase in and prolongation of the hyperalgesic response to a subthreshold or mild noxious stimulus [32,157,158]. Other studies have shown that animals exposed to stressors also exhibit changes in the spinal cord. In particular, animals showed a greater expression of c-fos in response to formalin, as well as a reduction in the basal and induced release of the inhibitory neurotransmitter GABA; a reduction in mu-opioid agonist antinociception enhanced the basal and evoked the release of glutamate [159,160], suggesting both increased central excitability and reduced central inhibition. In animals, stress-induced hyperalgesia was reduced by the spinal blockade of substance P, calcitonin gene-related peptide (CGRP), NMDA-glutamate receptors and neurokinin-1 receptors, all substances involved in the neurotransmission of pain [158]. At the supraspinal level, cold stress-induced alterations were observed in the serotonergic system, with reductions in both serotonin (5-HT) and 5-hydroxy indoleacetic acid (5-HIAA) levels in the supraspinal regions [161]. Thus, stress and psychological factors are involved in the development and severity of FM.

Sleep disorders are classically described within the symptomatic process of fibromyalgia. However, some recently reported data have generated the hypothesis that such disorders may be included among the causative factors of this pathology, rather than among its manifestations. Studies published in recent years have described a bidirectional correlation between sleep disturbances and widespread musculoskeletal pain, and it even seems that insomnia tends to precede the onset of pain and has predictive value regarding its onset and its persistence [162,163]. Studies carried out in healthy subjects also seem to show that total, partial and stage-specific sleep deprivation leads to hyperalgesia, an increased incidence of spontaneous pain and mood alterations, particularly anxiety and depression [164,165]. In a further study by Smith et al., the authors hypothesized that the development or aggravation of somatic and psychiatric symptoms is secondary to sleep discontinuity rather than sleep deprivation [166]. In addition to the number of awakenings, the cyclic alternating pattern (CAP) is a useful tool to analyze this discontinuity. It is represented by short cycles of periodic electroencephalographic activity of non-REM sleep, distinct from the background rhythm and with a periodicity of up to one minute [167]. CAP has been shown to be frequent in fibromyalgia patients and correlated with poor quality of sleep and with the severity of pain observed in these patients [168]. 

Findings in human studies carried out through the application of evoked potentials indicate that increased nociceptive sensitivity in response to sleep deprivation could derive from dysregulation of the descending pathways of pain control or from cognitive amplification of the central origin, thus excluding the mechanism of sensory amplification [169]. Biochemical analyses suggest that an insufficient amount of sleep could also play a facilitating role in nociception through the elevation of the serum concentration of IL-6, thus entering the pathogenetic cascade with an inflammatory substrate [164]. The structural analysis of sleep obtained by EEG studies provides additional support for the hypothesis that sleep alterations are among the causative factors of fibromyalgia. One of the first works on this aspect was a study by Moldofsky et al., in which microstructural analysis identified the presence of a rhythm component typical of wakefulness within the non-REM sleep pattern, particularly during periods of slow delta rhythm (0.5 to 2 Hz, characteristic of deep sleep), among both fibromyalgia patients and healthy subjects deprived of the deeper stages of alpha sleep (8 to 13 Hz). Moreover, in healthy subjects, deprivation was accompanied by a set of musculoskeletal and psychological symptoms similar to those chronically reported by patients. In light of these data, a hypothesis was put forward that considered fibromyalgia, then called fibrositis, to be a “non-restorative sleep syndrome”, in which an arousal mechanism (presumably responsible for the alpha component) interferes with non-REM sleep and its restorative function, consequently generating mood alterations and characteristic somatic disturbances [170]. More recent human studies have described the mechanisms underlying alpha-delta sleep (ADS, the intrusion of alpha rhythms in the deep phases of sleep), highlighting the role played by the thalamus, which, in turn, is modulated by GABAergic and cholinergic afferents [171,172]. It has been observed that the ADS phenomenon manifests itself in three different patterns: phasic (contemporary with delta activity), tonic (continuous throughout NREM sleep) and low alpha activity. Among these, the phasic pattern appears to be the most common among fibromyalgia patients and is the one that correlates most strongly with symptoms such as insomnia and pain [173]. It should be noted, however, that alpha-delta sleep is not an exclusive feature of fibromyalgia: it is also seen in a number of chronic pain syndromes and in some healthy individuals. Unlike alpha activity, the functional anatomical substrate of sleep spindles has been extensively studied. These are trains of electroencephalographic waves with a frequency between 12 and 16 Hz, lasting between 0.5 and 1.5 s and recurring every 3 to 10 s, and characteristic of non-REM (NREM) sleep, particularly the N2 stage of sleep (intermediate sleep). Sleep spindles are generated by the rhythmic firing of thalamic relay neurons, and their role is central in the induction and maintenance of NREM sleep, as well as in the gating mechanism through which transmission and the consequent cortical response to both internal and external stimuli are attenuated during sleep (control of arousal status) [174,175,176,177]. The frequency of spindles during NREM sleep is modulated by a series of factors, including age (inverse proportionality) and a certain degree of interindividual variability, as well as various pathological conditions in the neuropsychiatric field, such as depression, anxiety and stress [178]. A study by Landis et al. described a reduction in the frequency and amplitude of sleep spindles in a population of women with fibromyalgia compared to a control group, proposing the hypothesis of a dysfunction of the thalamo-cortical circuits underlying this alteration [179]. In a rat study, a deep learning method, known as SpindleNet, was applied to characterize sleep spindle activity in animals with induced chronic pain [180]. The results showed a correlation between a decrease in the frequency of spindles during NREM sleep and the level of chronic pain and allodynia, suggesting that this finding could be a biomarker of chronic pain as well as a target for neuromodulator therapy [181]. It is therefore possible to hypothesize that a dysfunctional primitive thalamus causes an alteration in its spindle pacemaker activity and alpha activity, compromising the restorative function of sleep and consequently generating the somatic and psychological symptoms of fibromyalgia, similar to what was suspected by Moldofsky et al. In light of the important function of the thalamus in sensory transmission pathways, it can also be hypothesized that both sleep disturbances and hyperalgesia/allodynia are the direct result of thalamic alteration, representing independent manifestations of the same pathological process. The relationship between sleep disturbances and fibromyalgia has not yet been fully clarified, and new studies will be needed to better define the relationship between the two. At the moment, the main hypotheses converge in their proposal of a bidirectional correlation characterized by a positive feedback circuit.

## 3. Pain Amplification in FM

Peripheral impulses are transmitted to the central nervous system by myelinated Aδ and unmyelinated C fibers. Pain signals travel through Aδ fibers very rapidly (~10 m/s) to the central nervous system, while those mediated by C fibers travel relatively slowly (~1.6 m/s) [182]. Numerous clinical studies performed in patients with FM have shown that small unmyelinated C fibers and myelinated A fibers are both involved in peripheral neuroinflammation in FM [183,184]. Both Aδ and C fibers are found mainly in superficial organs, such as the skin. On the other hand, deep somatic structures, such as muscles and joints, are mainly supplied by C fibers. Aδ fibers are activated by thermal or mechanical stimuli and generally cause a pain sensation of short duration. However, C-fiber activation is caused by thermal, mechanical or chemical stimuli, which often result in poor localization and a widespread pain sensation typical of FM [185]. In addition to this direct path from the periphery to the spinal cord, antidromic propagation of the impulse also occurs, whereby the impulse is sent back to junction points towards the terminal of the C fiber, with the consequent release of pro-inflammatory substances [186,187]. C fibers, therefore, drive the release of pro-inflammatory cytokines and chemokines and neuropeptides; conversely, A fibers generally respond to these stimuli. This leads to a greater sensitivity of the neuron to second stimuli with the amplification of reactivity. This phenomenon is called peripheral sensitization. It was also observed that the function of C fibers is influenced not only by local factors but also by systemic ones. Explaining multifactorial diseases such as FM appears to be very difficult with the linear reductionist medical model. In this regard, a more coherent picture can be derived from paradigms originating from the theory of complexity. FM is thought to represent the degradation of the autonomic nervous system in a failed attempt to adapt to a hostile environment. Complex control systems are known to have fractal structures, and fractal heart rate variability (HRV) reflects the performance of the autonomic nervous system. Therefore, a group of researchers attempted to measure the fractal scaling index of HRV in subjects with FM and correlate this value with clinical symptoms. The results of this study reveal that the alpha-1 fractal exponent of the heart rate is altered in patients with FM. This suggests a rigidity in the performance of the autonomic nervous system, thus supporting the idea that FM represents the degradation of the main complex adaptive nervous system [188,189].

In addition, animal studies have shown that this nervous system can modulate both peripheral neurons and innate immune cells, keratinocytes and dendritic cells by overexpressing α1-adenoreceptors [190,191]. The link between the peripheral consequences of sympathetic hyperactivity and central neuroinflammation has not yet been elucidated. For example, elevated levels of IL-8 but not IL-1β are observed in the CSF of FM patients, suggesting central neuroinflammation [106].

Other findings that have been observed in approximately 50% of FM patients are abnormalities in the morphology, neurophysiology or function of both myelinated small Aδ fibers and unmyelinated C fibers [73,75]. These data have been confirmed by studies that used microneurography to demonstrate structural changes in C fibers and the associated Schwann cells [76,192]. Another association that has been noted in FM is between miR-let-7d microRNA and a low density of nerve fibers. Furthermore, the aberrant expression of miR-let-7d and insulin-like growth factor 1 was observed in the skin of patients with FM who had alterations in their small fibers [193]. The underlying cause of all these results concerning small peripheral fibers is not clear, but it seems to be related to the consequences of local neurogenic inflammation owing to the effects that inflammatory products have on these sensory fibers.

Furthermore, in vivo, a decrease in the density of peripheral nerve fibers of the hind leg was demonstrated in a rat model of FM, which was induced by increasing glutamate levels [194]. This shows that changes in small fibers are related to alterations occurring in the brain in a top-down process. An important factor in this mechanism appears to be a reduction in the activity of descending antinociceptive pathways that interact and modulate pain-transmitting neurons of the activated dorsal horn. Typically, these pathways go from the subcortical structures down to the spinal cord. Furthermore, they are normally active and inhibit the upward transmission of the pain signal. When this inhibition is reduced, the pain sensation is amplified. Abnormal and higher pain processing is also observed at the CNS level [195,196]. A similar mechanism could also be involved in humans. In this regard, clinical studies have shown that the increase in nociceptive activity in muscles and other tissues following neurogenic inflammation could further contribute to central sensitization through a greater non-cyclical input into the spinal cord [20,197].

## 4. Diagnostic Biomarkers

The diagnosis of FM is currently based only on a complete clinical evaluation; until 2010, it relied on the 1990 ACR criteria [198] of widespread pain, with at least three consecutive months of pain and [199] “pain points” with digital palpation. New ACR criteria have been used since 2010 and are based on two new parameters: the diffuse pain index and the score on the symptom severity scale, both somatic and cognitive [9]. Tender points and the algometric measure of the pressure pain threshold are still important factors for a complete musculoskeletal clinical examination and for the exclusion of other diagnoses [200]. In 2016, the previous criteria were revisited to decrease the probability of an incorrect FM diagnosis [10]. However, individual phenotypic variability and the coexistence of other pathologies can lead to clinical examinations that are inadequate for a precise diagnosis, making it impossible to decide on universal criteria for this disease. Furthermore, specific biomarkers do not yet exist, and the research is therefore directed towards studying new indicators for the objective diagnosis of affected individuals through the identification of the genetic, environmental and epigenetic factors underlying the physiopathology of FM [201,202] (Table 1). 

### 4.1. Genetic Approach

The prevalence of fibromyalgia in family clusters and several studies supports the theory that genetic factors, in conjunction with environmental causes such as trauma, illnesses or emotional stress, could predispose individuals to FM. Key gene polymorphisms that are regarded as risk factors for fibromyalgia are those that are involved in mood disorders, although some findings are controversial. These candidate genes include the serotonin transporter (5-HTT), the serotonin 2A (5-HT2A) receptor, catechol-O-methyltransferase (COMT) and the dopamine receptor. 

A higher frequency of the S/S genotype of the 5-HTT gene was observed in FM patients compared to in healthy subjects [205]. However, this association may be limited to subjects with concomitant affective disorders, as it has not been confirmed in FM patients without depression or anxiety [206]. A reduction in the dopamine D4 receptor gene was also observed in FM patients [227]. With regard to COMT, homozygous low activity (Met/Met) and heterozygous low activity (Val/Met) genotypes were predominant in FM patients, while the homozygous high activity (Val/Val) genotype was less frequent [228]. In particular, the Met/Met genotype appears to be associated with greater disease severity in the domains of pain, fatigue, sleep disturbances and stress. In addition, compared to those with the Val/Met or Val/Val genotype, patients with the Met/Met polymorphism reported a greater decline in a positive attitude on days when the pain was greater [229]. A lower frequency of the 118 G allele of the μ1 opioid receptor gene was also found in patients with FM [212]. Furthermore, FM and its severity appear to be associated with various polymorphisms of the adrenergic receptor gene [230].

Other genes that have been associated with FM and that regulate nociceptive and analgesic neuronal pathways are the receptor 1 gene (*TAAR1*), the G protein signal 4 regulator gene (*RGS4*), the cannabinoid receptor 1 gene (*CNR1*) and the ionotrophic glutamate receptor gene AMPA 4 (*GRIA4*) [211]. 

Genome-wide association studies aiming to identify genes potentially involved in the pathogenesis of fibromyalgia have reported that genetic factors may be responsible for up to 50% of vulnerability to the disorder. *SLC64A4, TRPV2, MYT1L* and *NRXN3* are possible candidate genes found to be related to fibromyalgia. The frequency of polymorphisms in selected metabolism genes such as CYP P450 in FM pathology was also reported in another study [218].

In addition, a gene–environment association involving epigenetic changes was suggested as a triggering mechanism: fibromyalgia appears to be particularly characterized by a hypomethylated DNA pattern in genes involved in the stress response, DNA repair, the autonomic system response and subcortical neuronal abnormalities. In multiple tissues, differences were observed in the genome-wide expression profile of microRNAs, suggesting the involvement of various processes in the pathogenesis of fibromyalgia. Single nucleotide polymorphisms (SNPs) have been identified as possible candidates that are directly linked to FM susceptibility. 

### 4.2. Epigenetic Modifications

Previous studies have shown that early life experience and environmental factors in general may modulate genome function and the phenotype through epigenetic mechanisms without changing the DNA sequence [231]. In chronic pain, epigenetic pathways have been shown to play a significant role in mediating long-term changes in the central and peripheral nervous systems [232]. In particular, changes in the state of methylation, histone modifications and the expression of miRNAs seem to arise in the presence of peripheral inflammation and nerve damage in pain-related regions [233,234]. As a valuable diagnostic method, epigenetic modifications (such as DNA methylation) should be further investigated. The *BDNF, NAT15, HDAC4, PRKCA, RTN1* and *PRKG1* genes were mapped to differently methylated sites, indicating the potential involvement of FM nervous system development, skeletal/organ system development and chromatin compaction pathways. Differentially methylated sites that correlated with the FM map were frequently identified in genes involved in biological functions such as DNA repair, immune response and membrane transport genes.

### 4.3. MicroRNAs as Novel Possible Biomarkers

At least 30 percent of human genes are regulated by microRNAs [235], each of which can repress hundreds of genes [236]. The existence of microRNAs in various cellular compartments and their stability in the extracellular environment make them promising biomarkers to better understand the etiology of complex diseases such as FM.

Argonaute proteins may be packaged with miRNAs in exosomes and transported in biological fluids. In chronic pain conditions, miRNAs were observed to play a fundamental role [237], in which they modify the expression of signaling molecules, transmitters, ion channels or structural proteins, leading to the production of long-term hyperexcitability in peripheral nociceptive neurons and CNS [238].

A genome-wide expression profile analysis of microRNAs in the CSF of females with FM [239] demonstrated a relationship between human miRNAs and unusual FM symptoms, and only miR-145-5p was strongly linked to pain and fatigue in FM patients. Another study revealed a six- to 13-fold inhibition of five miRNAs—miR-451a, miR-338-3p, miR-143-3p, miR-145-5p and miR-223-3p—in FM patients compared to controls [239]. Masotti et al. [240] specifically selected FM patients for their study: the expression of six miRNAs (miR-23a-3p, miR-1, miR-133a, miR-346, miR-139-5p and miR-320b) was downregulated in FM patients in comparison with controls. Interestingly, miR-23a was downregulated in both the CSF and serum of patients with FM, although it was not significantly correlated with FM symptoms [239]. 

These polymorphisms are also correlated with psychiatric conditions; thus, they may be linked to psychiatric comorbidities instead of fibromyalgia alone. In addition, genetic findings are always ambiguous, and fibromyalgia has not been closely related to any single candidate gene.

### 4.4. Gene Expression

Since gene expression is modulated by epigenetic pathways, studies have compared transcriptional changes between FM patients and controls. A previous study identified 482 genes differentially expressed between patients and healthy controls, and the results indicated a relationship between FM status and the upregulation of inflammatory cytokine genes (*IL10, IL25* and *1L36A*) [226]. In addition, several genes in the solvent solute carrier family were found to be upregulated in FM subjects, including *SLC1A5* and *SLC25A22*, which encode glutamate transporters in the CNS [241]. In FM subjects, the metabotropic glutamate receptor (*GRM6*) gene encoding the group III G protein-coupled receptor, which is associated with the inhibition of the cyclic AMP cascade and involved in neuropathic pain signaling in dorsal horn neurons, was also upregulated [242]. The alteration of these paths [241,242] may be important in FM pathogenesis and must therefore be validated in a broad, separate, multicenter cohort of subjects with higher clinical heterogeneity. Furthermore, no studies have examined whether the observed changes in gene expression represent epigenetic mechanisms.

### 4.5. Mu-Opioid Receptor on B Lymphocytes as a Biomarker

It is important to note that the endogenous opioid system is similar to the immune system due to the presence of opioid receptors on the lymphocyte membrane. Among these receptors, the Mu-opioid receptor on B lymphocytes has been proposed as a specific biomarker for FM patients. The results obtained from a recent study showed a lower percentage of Mu-positive B cells in subjects with FM than in controls. Therefore, this receptor could be used as a biomarker for the objective diagnosis of patients who report chronic pain, such as that perceived by patients with FM [243].

## 5. Serological Markers

There is considerable interest in using a simple blood test to diagnose fibromyalgia. Therefore, several attempts have been made to detect unique serological markers. These findings, as well as those of genetic testing, are frequently contradictory, and no clinical tests have yet been confirmed to date (Table 2).

### 5.1. Autoantibodies

The association between antipolymer antibody (APA) and fibromyalgia was assessed because APAs were present in the serum of women with fibromyalgia-like signs [264]. One study analyzed the serum APA concentration of fibromyalgia patients, tension-type headache patients and safe controls. APAs were found in just 17.6% of fibromyalgia patients, confirming the lack of diagnostic significance of APA [265]. Two other autoantibodies—anti-68/48 kDa and anti-45 kDa—have been identified as potential markers for certain clinical subsets of primary fibromyalgia and chronic fatigue syndrome, as well as for secondary fibromyalgia/psychiatric disorders [245]. Antiserotonin, antiganglioside and antiphospholipid antibodies have been shown to be higher in patients with fibromyalgia as well as those with chronic fatigue syndrome, supporting the hypothesis that fibromyalgia and chronic fatigue syndrome belong to the same clinical entity and manifest themselves as “psycho-neuro-endocrinological autoimmune disorders” [266]. Another group investigated the prevalence and possible diagnostic significance of antiserotonin, antithromboplastin and antiganglioside in fibromyalgia patients. The authors reported an elevated prevalence of serotonin and thromboplastin antibodies but concluded that they had no diagnostic relevance [267]. Later, the association between thyroid autoimmunity and fibromyalgia was confirmed [268]. Pamuk and Cakir also reported a higher frequency of thyroid autoimmunity in fibromyalgia patients [269]. The correlation between rheumatic and thyroid disorders has long been recognized, with rheumatoid arthritis, Sjögren’s syndrome and systemic lupus erythematosus being the most prominent associations. In a subsequent work, Bazzichi et al. [270] also observed that the occurrence of thyroid disease aggravated FM symptoms. In particular, FM comorbidity was assessed in patients with Hashimoto’s thyroiditis (HT) with or without subclinical hypothyroidism (SCH) and in patients with SCH alone [270]. Other authors who studied the role of the thyroperoxidase antibody (TPO Ab) have also reported results that implicate the thyroid in the etiopathogenesis of FM [271]. A high prevalence of thyroid autoantibodies in FM patients has been shown in repeated studies, but the role of TPO Ab in FM remains unclear [271]. Therefore, the proposed antibodies have not yet been validated as potent, useful diagnostic biomarkers for fibromyalgia, and further studies are required. The measurement of amino acids in the plasma of FM patients is another area of interest. In the plasma of FM patients, Maes et al., found substantially lower levels of branched-chain amino acids, and they hypothesized that this deficiency could lead to a lack of energy supply and altered protein synthesis in the muscles [272]. In addition, an Italian research group also reported improvements in the levels of many amino acids in FM patients [273]. The plasma levels of 20 amino acids from 34 FM patients were analyzed, and significantly lower levels of taurine, alanine, tyrosine, valine, methionine, phenylalanine and threonine were found; these results may suggest the potential intestinal malabsorption of the amino acids mentioned [273].

### 5.2. Neuropeptides

Neuropeptide Y has been shown to both decrease and induce pain. Crofford and his colleagues were the first to test plasma neuropeptide Y levels in patients with fibromyalgia and found that they were substantially lower in the analyzed patients than in normal subjects [131]. These results are inconsistent with Anderberg’s subsequent findings of substantially elevated plasma levels of neuropeptide Y in patients with fibromyalgia relative to healthy subjects [274]. In addition, the serum levels of neuropeptide Y were shown to be substantially higher in subjects with fibromyalgia than in healthy controls in two different studies [249,265]. Another neuropeptide studied extensively in fibromyalgia patients and mice is substance P [275,276]. The results of one study suggested that substance P caused sleep disturbances in mice [276]. Subsequently, an elevated level of substance P was reported to be a likely cause of sleep disturbances in fibromyalgia patients [275,276].

### 5.3. BDNF

Some studies have also indicated the involvement of BDNF in pain syndromes [252]. Based on these data, Laske and collaborators were the first to examine serum BDNF concentrations in patients with fibromyalgia and found that their levels were dramatically increased compared to healthy controls. Two separate groups observed substantially higher levels of BDNF in the plasma and cerebral spinal fluid of fibromyalgia patients than in controls, suggesting the involvement of BDNF in fibromyalgia pathophysiology [220,277]. 

### 5.4. Glutamate

Compared to healthy controls, fibromyalgia patients displayed higher levels of glutamate compounds in the right amygdala, and pain was associated with elevated levels of glutamate in the left thalamus [278]. These results have implications for possible therapies directed against glutamate receptors; however, more research is desirable to determine whether these findings are applicable to other functional pain syndromes.

### 5.5. Inflammatory Cytokines

Cytokines have been suggested to be involved in fibromyalgia syndrome, but the findings tend to be controversial, particularly because there have been reports of increases, decreases and no substantial changes. It has been repeatedly shown that IL-8, a pro-inflammatory cytokine, is increased in fibromyalgia patients. Given that IL-8 promotes sympathetic pain, it may play an important role in the frequency of pain in fibromyalgia. The IL-8 expression pattern could aid in the diagnosis of fibromyalgia and in effective treatment strategies if confirmed in further studies [271]. 

### 5.6. Proteomic Approach

It has become generally accepted in the last few years that the genome represents only the first layer of complexity. Biological functions are dependent on a complex protein population, and protein characterization can reveal posttranslational changes (e.g., phosphorylation, glycosylation and methylation) and provide insight into protein–protein interactions and functions. For this reason, the field of proteomics, which is the identification of proteins in biological samples such as body fluids and tissue extracts, is receiving growing interest. Whole saliva analysis was performed to measure the protein content and to evaluate quantitative or qualitative differences between patients with fibromyalgia and healthy subjects [279]. For example, compared to healthy subjects, cyclophilin A was overexpressed in fibromyalgia patients [279]. Other proteins found to be overexpressed in fibromyalgia were calgranulins, belonging to the S100 multigene family, which is involved in a range of intracellular activities, such as cell proliferation and differentiation, cytoskeletal interactions, membrane rearrangement and structural organization, intracellular Ca^2+^ homeostasis, cell migration, inflammation and defense against oxidative cell damage [279]. Transaldolase and phosphoglycerate mutase 1 were also found to be substantially overexpressed in fibromyalgia patients relative to their levels in controls [279]. Researchers observed, however, that several proteins were expressed differently: calgranulin A, calgranulin C, cyclophilin A, profilin 1, Rho guanosine diphosphate (GDP)-dissociation inhibitor 2, proteasome subunit-a-type-2 and haptoglobin-associated protein precursor [279]. These proteins play important roles in the immune response, the remodeling of the cytoskeleton and the inflammatory process, but their role in FM continues to be controversial. Furthermore, serum proteome profiles revealed dysregulated proteins and pathways associated with fibromyalgia syndrome in women. Upregulated inflammation, as observed in the study of serum proteomics, plays a major role in the pathogenesis of FM [257]. Combining METTL18, IGLV3-25, IL1RAP and IGHV1OR21-1 levels can successfully distinguish FM patients from pain-free controls. In the future, differentially expressed proteins could serve as potential biomarkers for FM diagnosis and clinical assessment [257].

### 5.7. Metabolomic Approach

In an untargeted ^1^H NMR metabolomics examination of urine samples, a group of clinically well-defined female FM patients with no psychiatric co-morbidities could be completely distinguished from a group of young healthy women [261]. The presence of metabolic markers of disturbances (hippuric, 2-hydroxyisobutyric and lactic acids) in the gut microbiome supports the paradigm that control of the gut–brain axis is impaired by stress-related disorders such as FM. For the distinction between FM patients and controls, three metabolite markers (taurine, creatine and succinic acid) were important and were also effective measures of pain and fatigue symptoms in FM syndrome [261]. However, further studies are still needed.

## 6. Antioxidants and Diet for Fibromyalgia Management

Fibromyalgia patients produce higher levels of harmful free radicals than healthy people and have a decreased antioxidant ability, contributing to oxidative stress. Compared to other areas of the body, the central nervous system is highly susceptible to ROS because of its high lipid content [280]. The progression of fibromyalgia may be dependent on increased ROS. Treatment with antioxidants and vitamins, in addition to antidepressants and structural analogs of gamma-aminobutyric acid, was able to change the florid symptoms of FM patients [281]. Certain groups of bioactive compounds derived from medicinal plants have also demonstrated analgesic activity and antioxidant properties with respect to FM: these include essential oils [282], extracts [283], monoterpenes [284], sesquiterpenes [285] and alkaloids [286] (Table 3).

## 7. Conclusions

FM is a complicated syndrome characterized by chronic pain, joint rigidity, fatigue, sleep interruption, cerebral dysfunction and depression. Research on FM is becoming progressively more significant because of the compromised quality of life of patients and the economic weight on the medical care system. The pathogenesis of fibromyalgia is not well known, and the diagnosis is only clinical at present. Oxidative stress, mitochondrial dysfunction, multivitamin deficiencies and a disproportion between oxidants and antioxidants are interesting and clinically attractive topics that require further study to clarify the state and development of FM.

To date, no objective tests or biomarkers with sufficient diagnostic accuracy have been identified, and current analyses can only indicate a predisposition to fibromyalgia. Numerous studies, however, provide insights into the pathophysiology of fibromyalgia. Proteomic research as well as gene expression profiling may have potential applications as novel methods for the diagnosis of FM. 

Pharmacological treatment alone is inadequate for the majority of patients who suffer from FM syndrome. Given the different mechanisms of pain sensitivity, treatments will continue to involve multidisciplinary programs that target the peripheral, central, cognitive-emotional and interpersonal causes of the chronic pain that characterizes FM pathophysiology.

## Figures and Tables

**Table 1:** Candidate genes in the pathogenesis of FM [202].

Gene	Type of Study	References
5-HTT	Human	[203,204,205,206]
COMT	Human	[207,208,209,210]
TAAR1	Human	[211]
Opioid receptor μ1 gene A118G	Human	[212]
RGS4	Human	[121,211]
CNR1	Human	[211,213]
GRIA4	Human	[211,213,214]
SLC64A4	Human	[215,216]
TRPV2	Human	[214,215,217]
MYT1L	Human	[214]
NRXN3	Human	[125,215]
CYP450	Human	[218]
BDNF	Human	[219,220,221,222,223]
NAT15	Human	[224]
HDAC4	Human	[224]
PRKCA	Human	[224,225]
RTN1	Human	[224]
PRKG1	Human	[224]
SLC1A5	Human	[226]
SLC25A22	Human	[226]
GRM6	Human	[226]

**Table 2:** Blood markers for FM diagnosis.

Markers	Type of Study	References
Classic autoantibodies (SS-A/Ro, SS-B/La, ANA, and RF) Specific autoantibodies (SP-1, CA6, PSP)	Human	[244,245,246,247,248]
Neuropeptides	Human	[249,250,251]
BDNF	Human	[220,252]
Glutamate	Human	[253,254]
Inflammatory cytokines	Human	[102,255,256]
Proteomic analysis	Human	[257]
Metabolomic analysis	Human	[258,259,260,261,262,263]

**Table 3:** Compounds with antioxidant and analgesic properties for FM management.

Compound	Effects	References
Melatonin	In an animal study, melatonin was able to improve behavioral defects, oxidative and nitrosative stress, mast cell infiltration and activation of microglia in a reserpine-induced FM model.	[287]
	In a clinical trial, the exogenous administration of 10 mg of melatonin once every 24 h increased endogenous pain inhibition, assessed on a numerical scale (0–10). The combination of amitriptyline and melatonin provided better results than amitriptyline alone, as calculated by the visual analog pain scale, in subjects with FM.	[288]
	A randomized trial found that melatonin alone or in combination with fluoxetine was beneficial for the treatment of FM. Using melatonin (3 or 5 mg/day) in combination with 20 mg/day fluoxetine caused a significant reduction in both total and individual components of the Fibromyalgia Impact Questionnaire score compared to the pretreatment values.	[289]
Coenzyme Q10	CoQ10 treatment showed effects on clinical symptoms, blood mononuclear cells and markers of mitochondrial and oxidative stress in women with FM.	[290]
	The results of this clinical study suggest that CoQ10 supplementation plays a role in the modulation of mitochondrial dysfunction and oxidative stress that induce headaches in individuals with FM.	[291]
	In a clinical study, CoQ10 supplementation was shown to provide additional benefits for relieving pain sensation in FM patients treated with pregabalin, possibly by improving mitochondrial function, reducing inflammation and decreasing brain activity.	[292]
Vitamins D and E	A clinical study found that women with FM had a lower qualitative and quantitative intake than control subjects. In particular, an association has been found between vitamin D deficiency and FM. However, its role in FM pathophysiology and the clinical relevance of its identification and treatment requires further clarification. Only vitamin E appears to be related to quality of life and pain sensation.	[117,293,294]
Palmitoylethanolamide (PEA)	PEA is a major anti-inflammatory, analgesic and neuroprotective mediator in central and peripheral organs and systems and acts on several molecular targets.	[295,296]
	PEA is emerging as a candidate biomarker due to its anti-inflammatory and anti-hyperalgesic effects via the downregulation of mast cell activation. Preclinical and clinical studies support the idea that PEA merits further consideration as a therapeutic approach for controlling inflammatory responses, pain, related peripheral neuropathic pain and symptoms of FM.	[297,298,299,300,301,302,303,304,305,306]

## Data Availability

Not applicable.

## References

[1] Gerdle B., Bjork J., Coster L., Henriksson K., Henriksson C., Bengtsson A. (2008). Prevalence of widespread pain and associations with work status: A population study. BMC Musculoskelet. Disord..

[2] Bennett R.M., Jones J., Turk D.C., Russell I.J., Matallana L. (2007). An internet survey of 2596 people with fibromyalgia. BMC Musculoskelet. Disord..

[3] Bellato E., Marini E., Castoldi F., Barbasetti N., Mattei L., Bonasia D.E., Blonna D. (2012). Fibromyalgia syndrome: Etiology, pathogenesis, diagnosis, and treatment. Pain Res. Treat..

[4] Gowers W.R. (1904). A Lecture on Lumbago: Its Lessons and Analogues: Delivered at the National Hospital for the Paralysed and Epileptic. Br. Med. J..

[5] Graham W. (1953). The fibrosits syndrome. Bull. Rheum. Dis..

[6] Smythe H.A., Moldofsky H. (1977). Two contributions to understanding of the “fibrositis” syndrome. Bull. Rheum. Dis..

[7] Staud R., Vierck C.J., Cannon R.L., Mauderli A.P., Price D.D. (2001). Abnormal sensitization and temporal summation of second pain (wind-up) in patients with fibromyalgia syndrome. Pain.

[8] Wolfe F. (2010). New American College of Rheumatology criteria for fibromyalgia: A twenty-year journey. Arthritis Care Res..

[9] Wolfe F., Clauw D.J., Fitzcharles M.A., Goldenberg D.L., Katz R.S., Mease P., Russell A.S., Russell I.J., Winfield J.B., Yunus M.B. (2010). The American College of Rheumatology preliminary diagnostic criteria for fibromyalgia and measurement of symptom severity. Arthritis Care Res..

[10] Wolfe F., Clauw D.J., Fitzcharles M.A., Goldenberg D.L., Hauser W., Katz R.L., Mease P.J., Russell A.S., Russell I.J., Walitt B. (2016). 2016 Revisions to the 2010/2011 fibromyalgia diagnostic criteria. Semin. Arthritis Rheum..

[11] Wolfe F., Ross K., Anderson J., Russell I.J., Hebert L. (1995). The prevalence and characteristics of fibromyalgia in the general population. Arthritis Rheum..

[12] Bhargava J., Hurley J.A. (2021). Fibromyalgia. StatPearls.

[13] Meyer H.P. (2002). Myofascial pain syndrome and its suggested role in the pathogenesis and treatment of fibromyalgia syndrome. Curr. Pain Headache Rep..

[14] Clauw D.J. (2015). Fibromyalgia and related conditions. Mayo Clin. Proc..

[15] Malatji B.G., Mason S., Mienie L.J., Wevers R.A., Meyer H., van Reenen M., Reinecke C.J. (2019). The GC-MS metabolomics signature in patients with fibromyalgia syndrome directs to dysbiosis as an aspect contributing factor of FMS pathophysiology. Metabolomics.

[16] Bradley L.A. (2009). Pathophysiology of fibromyalgia. Am. J. Med..

[17] Yunus M.B., Khan M.A., Rawlings K.K., Green J.R., Olson J.M., Shah S. (1999). Genetic linkage analysis of multicase families with fibromyalgia syndrome. J. Rheumatol..

[18] Muir W.W., Woolf C.J. (2001). Mechanisms of pain and their therapeutic implications. J. Am. Vet. Med. Assoc..

[19] Vierck C.J. (2006). Mechanisms underlying development of spatially distributed chronic pain (fibromyalgia). Pain.

[20] Staud R., Nagel S., Robinson M.E., Price D.D. (2009). Enhanced central pain processing of fibromyalgia patients is maintained by muscle afferent input: A randomized, double-blind, placebo-controlled study. Pain.

[21] Brosschot J.F. (2002). Cognitive-emotional sensitization and somatic health complaints. Scand. J. Psychol..

[22] Overmier J.B. (2002). Sensitization, conditioning, and learning: Can they help us understand somatization and disability?. Scand. J. Psychol..

[23] Jackson P.L., Meltzoff A.N., Decety J. (2005). How do we perceive the pain of others? A window into the neural processes involved in empathy. Neuroimage.

[24] Collado A., Gomez E., Coscolla R., Sunyol R., Sole E., Rivera J., Altarriba E., Carbonell J., Castells X. (2014). Work, family and social environment in patients with Fibromyalgia in Spain: An epidemiological study: EPIFFAC study. BMC Health Serv. Res..

[25] O’Brien A.T., Deitos A., Trinanes Pego Y., Fregni F., Carrillo-de-la-Pena M.T. (2018). Defective Endogenous Pain Modulation in Fibromyalgia: A Meta-Analysis of Temporal Summation and Conditioned Pain Modulation Paradigms. J. Pain.

[26] Harris R.E., Clauw D.J. (2012). Imaging central neurochemical alterations in chronic pain with proton magnetic resonance spectroscopy. Neurosci. Lett..

[27] Clauw D.J. (2014). Fibromyalgia: A clinical review. JAMA.

[28] Sluka K.A., O’Donnell J.M., Danielson J., Rasmussen L.A. (2013). Regular physical activity prevents development of chronic pain and activation of central neurons. J. Appl. Physiol..

[29] Bobinski F., Ferreira T.A.A., Cordova M.M., Dombrowski P.A., da Cunha C., Santo C., Poli A., Pires R.G.W., Martins-Silva C., Sluka K.A. (2015). Role of brainstem serotonin in analgesia produced by low-intensity exercise on neuropathic pain after sciatic nerve injury in mice. Pain.

[30] Yokoyama T., Lisi T.L., Moore S.A., Sluka K.A. (2007). Muscle fatigue increases the probability of developing hyperalgesia in mice. J. Pain.

[31] Sluka K.A., Rasmussen L.A. (2010). Fatiguing exercise enhances hyperalgesia to muscle inflammation. Pain.

[32] Gregory N.S., Gibson-Corley K., Frey-Law L., Sluka K.A. (2013). Fatigue-enhanced hyperalgesia in response to muscle insult: Induction and development occur in a sex-dependent manner. Pain.

[33] Sharma N.K., Ryals J.M., Liu H., Liu W., Wright D.E. (2009). Acidic saline-induced primary and secondary mechanical hyperalgesia in mice. J. Pain.

[34] Kim S.H., Song J., Mun H., Park K.U. (2009). Effect of the combined use of tramadol and milnacipran on pain threshold in an animal model of fibromyalgia. Korean J. Intern. Med..

[35] Yokoyama T., Maeda Y., Audette K.M., Sluka K.A. (2007). Pregabalin reduces muscle and cutaneous hyperalgesia in two models of chronic muscle pain in rats. J. Pain.

[36] Sharma N.K., Ryals J.M., Gajewski B.J., Wright D.E. (2010). Aerobic exercise alters analgesia and neurotrophin-3 synthesis in an animal model of chronic widespread pain. Phys. Ther..

[37] Giesecke T., Gracely R.H., Grant M.A., Nachemson A., Petzke F., Williams D.A., Clauw D.J. (2004). Evidence of augmented central pain processing in idiopathic chronic low back pain. Arthritis Rheum..

[38] Gracely R.H., Geisser M.E., Giesecke T., Grant M.A., Petzke F., Williams D.A., Clauw D.J. (2004). Pain catastrophizing and neural responses to pain among persons with fibromyalgia. Brain.

[39] Wager T.D., Atlas L.Y., Lindquist M.A., Roy M., Woo C.W., Kross E. (2013). An fMRI-based neurologic signature of physical pain. N. Engl. J. Med..

[40] Segerdahl A.R., Mezue M., Okell T.W., Farrar J.T., Tracey I. (2015). The dorsal posterior insula subserves a fundamental role in human pain. Nat. Neurosci..

[41] Lee M.C., Tracey I. (2013). Imaging pain: A potent means for investigating pain mechanisms in patients. Br. J. Anaesth..

[42] Tracey I. (2013). “Seeing” how our drugs work brings translational added value. Anesthesiology.

[43] Eippert F., Tracey I. (2014). Pain and the PAG: Learning from painful mistakes. Nat. Neurosci..

[44] Napadow V., Kim J., Clauw D.J., Harris R.E. (2012). Decreased intrinsic brain connectivity is associated with reduced clinical pain in fibromyalgia. Arthritis Rheum..

[45] Napadow V., LaCount L., Park K., As-Sanie S., Clauw D.J., Harris R.E. (2010). Intrinsic brain connectivity in fibromyalgia is associated with chronic pain intensity. Arthritis Rheum..

[46] Jensen K.B., Loitoile R., Kosek E., Petzke F., Carville S., Fransson P., Marcus H., Williams S.C., Choy E., Mainguy Y. (2012). Patients with fibromyalgia display less functional connectivity in the brain’s pain inhibitory network. Mol. Pain.

[47] Harris R.E., Sundgren P.C., Craig A.D., Kirshenbaum E., Sen A., Napadow V., Clauw D.J. (2009). Elevated insular glutamate in fibromyalgia is associated with experimental pain. Arthritis Rheum..

[48] Fayed N., Garcia-Campayo J., Magallon R., Andres-Bergareche H., Luciano J.V., Andres E., Beltran J. (2010). Localized 1H-NMR spectroscopy in patients with fibromyalgia: A controlled study of changes in cerebral glutamate/glutamine, inositol, choline, and N-acetylaspartate. Arthritis Res..

[49] Harris R.E. (2010). Elevated excitatory neurotransmitter levels in the fibromyalgia brain. Arthritis Res..

[50] Harris R.E., Sundgren P.C., Pang Y., Hsu M., Petrou M., Kim S.H., McLean S.A., Gracely R.H., Clauw D.J. (2008). Dynamic levels of glutamate within the insula are associated with improvements in multiple pain domains in fibromyalgia. Arthritis Rheum..

[51] Harte S.E., Clauw D.J., Napadow V., Harris R.E. (2013). Pressure Pain Sensitivity and Insular Combined Glutamate and Glutamine (Glx) Are Associated with Subsequent Clinical Response to Sham but Not Traditional Acupuncture in Patients Who Have Chronic Pain. Med. Acupunct..

[52] Foerster B.R., Nascimento T.D., DeBoer M., Bender M.A., Rice I.C., Truong D.Q., Bikson M., Clauw D.J., Zubieta J.K., Harris R.E. (2015). Excitatory and inhibitory brain metabolites as targets of motor cortex transcranial direct current stimulation therapy and predictors of its efficacy in fibromyalgia. Arthritis Rheumatol..

[53] Harris R.E., Napadow V., Huggins J.P., Pauer L., Kim J., Hampson J., Sundgren P.C., Foerster B., Petrou M., Schmidt-Wilcke T. (2013). Pregabalin rectifies aberrant brain chemistry, connectivity, and functional response in chronic pain patients. Anesthesiology.

[54] Staud R., Vierck C.J., Robinson M.E., Price D.D. (2005). Effects of the N-methyl-D-aspartate receptor antagonist dextromethorphan on temporal summation of pain are similar in fibromyalgia patients and normal control subjects. J. Pain.

[55] Cohen S.P., Verdolin M.H., Chang A.S., Kurihara C., Morlando B.J., Mao J. (2006). The intravenous ketamine test predicts subsequent response to an oral dextromethorphan treatment regimen in fibromyalgia patients. J. Pain.

[56] Olivan-Blazquez B., Herrera-Mercadal P., Puebla-Guedea M., Perez-Yus M.C., Andres E., Fayed N., Lopez-Del-Hoyo Y., Magallon R., Roca M., Garcia-Campayo J. (2014). Efficacy of memantine in the treatment of fibromyalgia: A double-blind, randomised, controlled trial with 6-month follow-up. Pain.

[57] Holton K.F., Taren D.L., Thomson C.A., Bennett R.M., Jones K.D. (2012). The effect of dietary glutamate on fibromyalgia and irritable bowel symptoms. Clin. Exp. Rheumatol..

[58] Skyba D.A., Lisi T.L., Sluka K.A. (2005). Excitatory amino acid concentrations increase in the spinal cord dorsal horn after repeated intramuscular injection of acidic saline. Pain.

[59] Radhakrishnan R., Sluka K.A. (2009). Increased glutamate and decreased glycine release in the rostral ventromedial medulla during induction of a pre-clinical model of chronic widespread muscle pain. Neurosci. Lett..

[60] Skyba D.A., King E.W., Sluka K.A. (2002). Effects of NMDA and non-NMDA ionotropic glutamate receptor antagonists on the development and maintenance of hyperalgesia induced by repeated intramuscular injection of acidic saline. Pain.

[61] Da Silva L.F., Desantana J.M., Sluka K.A. (2010). Activation of NMDA receptors in the brainstem, rostral ventromedial medulla, and nucleus reticularis gigantocellularis mediates mechanical hyperalgesia produced by repeated intramuscular injections of acidic saline in rats. J. Pain.

[62] Da Silva L.F.S., Walder R.Y., Davidson B.L., Wilson S.P., Sluka K.A. (2010). Changes in expression of NMDA-NR1 receptor subunits in the rostral ventromedial medulla modulate pain behaviors. Pain.

[63] Lee H., Im J., Won H., Nam W., Kim Y.O., Lee S.W., Lee S., Cho I.H., Kim H.K., Kwon J.T. (2017). Effects of tianeptine on symptoms of fibromyalgia via BDNF signaling in a fibromyalgia animal model. Korean J. Physiol. Pharm..

[64] Messersmith D.J., Kim D.J., Iadarola M.J. (1998). Transcription factor regulation of prodynorphin gene expression following rat hindpaw inflammation. Brain Res. Mol. Brain Res..

[65] Anderson L.E., Seybold V.S. (2000). Phosphorylated cAMP response element binding protein increases in neurokinin-1 receptor-immunoreactive neurons in rat spinal cord in response to formalin-induced nociception. Neurosci. Lett..

[66] Wei F., Qiu C.S., Kim S.J., Muglia L., Maas J.W., Pineda V.V., Xu H.M., Chen Z.F., Storm D.R., Muglia L.J. (2002). Genetic elimination of behavioral sensitization in mice lacking calmodulin-stimulated adenylyl cyclases. Neuron.

[67] Ma W., Quirion R. (2001). Increased phosphorylation of cyclic AMP response element-binding protein (CREB) in the superficial dorsal horn neurons following partial sciatic nerve ligation. Pain.

[68] Miletic G., Pankratz M.T., Miletic V. (2002). Increases in the phosphorylation of cyclic AMP response element binding protein (CREB) and decreases in the content of calcineurin accompany thermal hyperalgesia following chronic constriction injury in rats. Pain.

[69] Hoeger-Bement M.K., Sluka K.A. (2003). Phosphorylation of CREB and mechanical hyperalgesia is reversed by blockade of the cAMP pathway in a time-dependent manner after repeated intramuscular acid injections. J. Neurosci..

[70] Chen W.K., Liu I.Y., Chang Y.T., Chen Y.C., Chen C.C., Yen C.T., Shin H.S., Chen C.C. (2010). Ca(v)3.2 T-type Ca^2+^ channel-dependent activation of ERK in paraventricular thalamus modulates acid-induced chronic muscle pain. J. Neurosci..

[71] Cheng S.J., Chen C.C., Yang H.W., Chang Y.T., Bai S.W., Chen C.C., Yen C.T., Min M.Y. (2011). Role of extracellular signal-regulated kinase in synaptic transmission and plasticity of a nociceptive input on capsular central amygdaloid neurons in normal and acid-induced muscle pain mice. J. Neurosci..

[72] Oaklander A.L., Herzog Z.D., Downs H.M., Klein M.M. (2013). Objective evidence that small-fiber polyneuropathy underlies some illnesses currently labeled as fibromyalgia. Pain.

[73] Uceyler N., Zeller D., Kahn A.K., Kewenig S., Kittel-Schneider S., Schmid A., Casanova-Molla J., Reiners K., Sommer C. (2013). Small fibre pathology in patients with fibromyalgia syndrome. Brain.

[74] Caro X.J., Winter E.F. (2014). Evidence of abnormal epidermal nerve fiber density in fibromyalgia: Clinical and immunologic implications. Arthritis Rheumatol..

[75] Doppler K., Rittner H.L., Deckart M., Sommer C. (2015). Reduced dermal nerve fiber diameter in skin biopsies of patients with fibromyalgia. Pain.

[76] Serra J., Collado A., Sola R., Antonelli F., Torres X., Salgueiro M., Quiles C., Bostock H. (2014). Hyperexcitable C nociceptors in fibromyalgia. Ann. Neurol..

[77] Staud R., Weyl E.E., Bartley E., Price D.D., Robinson M.E. (2014). Analgesic and anti-hyperalgesic effects of muscle injections with lidocaine or saline in patients with fibromyalgia syndrome. Eur. J. Pain.

[78] Srikuea R., Symons T.B., Long D.E., Lee J.D., Shang Y., Chomentowski P.J., Yu G., Crofford L.J., Peterson C.A. (2013). Association of fibromyalgia with altered skeletal muscle characteristics which may contribute to postexertional fatigue in postmenopausal women. Arthritis Rheumathol..

[79] Shang Y., Gurley K., Symons B., Long D., Srikuea R., Crofford L.J., Peterson C.A., Yu G. (2012). Noninvasive optical characterization of muscle blood flow, oxygenation, and metabolism in women with fibromyalgia. Arthritis Res..

[80] Dailey D.L., Keffala V.J., Sluka K.A. (2015). Do cognitive and physical fatigue tasks enhance pain, cognitive fatigue, and physical fatigue in people with fibromyalgia?. Arthritis Care Res..

[81] Law L.A.F., Sluka K.A., McMullen T., Lee J., Arendt-Nielsen L., Graven-Nielsen T. (2008). Acidic buffer induced muscle pain evokes referred pain and mechanical hyperalgesia in humans. Pain.

[82] Abdelhamid R.E., Sluka K.A. (2015). ASICs Mediate Pain and Inflammation in Musculoskeletal Diseases. Physiology.

[83] Sluka K.A., Gregory N.S. (2015). The dichotomized role for acid sensing ion channels in musculoskeletal pain and inflammation. Neuropharmacology.

[84] Deval E., Gasull X., Noel J., Salinas M., Baron A., Diochot S., Lingueglia E. (2010). Acid-sensing ion channels (ASICs): Pharmacology and implication in pain. Pharmacol. Ther..

[85] Molliver D.C., Immke D.C., Fierro L., Pare M., Rice F.L., McCleskey E.W. (2005). ASIC3, an acid-sensing ion channel, is expressed in metaboreceptive sensory neurons. Mol. Pain.

[86] Walder R.Y., Gautam M., Wilson S.P., Benson C.J., Sluka K.A. (2011). Selective targeting of ASIC3 using artificial miRNAs inhibits primary and secondary hyperalgesia after muscle inflammation. Pain.

[87] Karczewski J., Spencer R.H., Garsky V.M., Liang A., Leitl M.D., Cato M.J., Cook S.P., Kane S., Urban M.O. (2010). Reversal of acid-induced and inflammatory pain by the selective ASIC3 inhibitor, APETx2. Br. J. Pharm..

[88] Chen W.N., Chen C.C. (2014). Acid mediates a prolonged antinociception via substance P signaling in acid-induced chronic widespread pain. Mol. Pain.

[89] Gregory N.S., Brito R.G., Fusaro M., Sluka K.A. (2016). ASIC3 Is Required for Development of Fatigue-Induced Hyperalgesia. Mol. Neurobiol..

[90] Sluka K.A., Price M.P., Breese N.M., Stucky C.L., Wemmie J.A., Welsh M.J. (2003). Chronic hyperalgesia induced by repeated acid injections in muscle is abolished by the loss of ASIC3, but not ASIC1. Pain.

[91] Gautam M., Benson C.J., Ranier J.D., Light A.R., Sluka K.A. (2012). ASICs Do Not Play a Role in Maintaining Hyperalgesia Induced by Repeated Intramuscular Acid Injections. Pain Res. Treat..

[92] Taguchi T., Katanosaka K., Yasui M., Hayashi K., Yamashita M., Wakatsuki K., Kiyama H., Yamanaka A., Mizumura K. (2015). Peripheral and spinal mechanisms of nociception in a rat reserpine-induced pain model. Pain.

[93] Navratilova E., Porreca F. (2019). Substance P and Inflammatory Pain: Getting It Wrong and Right Simultaneously. Neuron.

[94] De Felipe C., Herrero J.F., O’Brien J.A., Palmer J.A., Doyle C.A., Smith A.J., Laird J.M., Belmonte C., Cervero F., Hunt S.P. (1998). Altered nociception, analgesia and aggression in mice lacking the receptor for substance P. Nature.

[95] Zimmer A., Zimmer A.M., Baffi J., Usdin T., Reynolds K., Konig M., Palkovits M., Mezey E. (1998). Hypoalgesia in mice with a targeted deletion of the tachykinin 1 gene. Proc. Natl. Acad. Sci. USA.

[96] Hill R. (2000). NK1 (substance P) receptor antagonists--why are they not analgesic in humans?. Trends Pharm. Sci.

[97] Lin C.C., Chen W.N., Chen C.J., Lin Y.W., Zimmer A., Chen C.C. (2012). An antinociceptive role for substance P in acid-induced chronic muscle pain. Proc. Natl. Acad. Sci. USA.

[98] Nugraha B., Karst M., Engeli S., Gutenbrunner C. (2012). Brain-derived neurotrophic factor and exercise in fibromyalgia syndrome patients: A mini review. Rheumatol. Int..

[99] Giovengo S.L., Russell I.J., Larson A.A. (1999). Increased concentrations of nerve growth factor in cerebrospinal fluid of patients with fibromyalgia. J. Rheumatol..

[100] Baumeister D., Eich W., Saft S., Geisel O., Hellweg R., Finn A., Svensson C.I., Tesarz J. (2019). No evidence for altered plasma NGF and BDNF levels in fibromyalgia patients. Sci. Rep..

[101] Slade G.D., Conrad M.S., Diatchenko L., Rashid N.U., Zhong S., Smith S., Rhodes J., Medvedev A., Makarov S., Maixner W. (2011). Cytokine biomarkers and chronic pain: Association of genes, transcription, and circulating proteins with temporomandibular disorders and widespread palpation tenderness. Pain.

[102] Sturgill J., McGee E., Menzies V. (2014). Unique cytokine signature in the plasma of patients with fibromyalgia. J. Immunol. Res..

[103] Mendieta D., De la Cruz-Aguilera D.L., Barrera-Villalpando M.I., Becerril-Villanueva E., Arreola R., Hernandez-Ferreira E., Perez-Tapia S.M., Perez-Sanchez G., Garces-Alvarez M.E., Aguirre-Cruz L. (2016). IL-8 and IL-6 primarily mediate the inflammatory response in fibromyalgia patients. J. Neuroimmunol..

[104] Littlejohn G., Guymer E. (2018). Neurogenic inflammation in fibromyalgia. Semin. Immunopathol..

[105] Bote M.E., Garcia J.J., Hinchado M.D., Ortega E. (2014). An exploratory study of the effect of regular aquatic exercise on the function of neutrophils from women with fibromyalgia: Role of IL-8 and noradrenaline. Brain Behav. Immunol..

[106] Kadetoff D., Lampa J., Westman M., Andersson M., Kosek E. (2012). Evidence of central inflammation in fibromyalgia-increased cerebrospinal fluid interleukin-8 levels. J. Neuroimmunol..

[107] Coskun Benlidayi I. (2019). Role of inflammation in the pathogenesis and treatment of fibromyalgia. Rheumatol. Int..

[108] Kosek E., Altawil R., Kadetoff D., Finn A., Westman M., Le Maitre E., Andersson M., Jensen-Urstad M., Lampa J. (2015). Evidence of different mediators of central inflammation in dysfunctional and inflammatory pain--interleukin-8 in fibromyalgia and interleukin-1 beta in rheumatoid arthritis. J. Neuroimmunol..

[109] Bote M.E., Garcia J.J., Hinchado M.D., Ortega E. (2012). Inflammatory/stress feedback dysregulation in women with fibromyalgia. Neuroimmunomodulation.

[110] Imamura M., Targino R.A., Hsing W.T., Imamura S., Azevedo R.S., Boas L.S., Tozetto-Mendoza T.R., Alfieri F.M., Filippo T.R., Battistella L.R. (2014). Concentration of cytokines in patients with osteoarthritis of the knee and fibromyalgia. Clin. Interv. Aging.

[111] Ranzolin A., Duarte A.L., Bredemeier M., da Costa Neto C.A., Ascoli B.M., Wollenhaupt-Aguiar B., Kapczinski F., Xavier R.M. (2016). Evaluation of cytokines, oxidative stress markers and brain-derived neurotrophic factor in patients with fibromyalgia–A controlled cross-sectional study. Cytokine.

[112] Mastrangelo F., Frydas I., Ronconi G., Kritas S.K., Tettamanti L., Caraffa A.C., DOvidio C., Younes A., Gallenga C.E., Conti P. (2018). Low-grade chronic inflammation mediated by mast cells in fibromyalgia: Role of IL-37. J. Biol. Regul. Homeost. Agents.

[113] Cicuttini F.M., Wluka A.E. (2016). Not just loading and age: The dynamics of osteoarthritis, obesity and inflammation. Med. J. Aust..

[114] Rossi H.L., Luu A.K., DeVilbiss J.L., Recober A. (2013). Obesity increases nociceptive activation of the trigeminal system. Eur. J. Pain.

[115] Rossi H.L., Luu A.K., Kothari S.D., Kuburas A., Neubert J.K., Caudle R.M., Recober A. (2013). Effects of diet-induced obesity on motivation and pain behavior in an operant assay. Neuroscience.

[116] Smart P.A., Waylonis G.W., Hackshaw K.V. (1997). Immunologic profile of patients with fibromyalgia. Am. J. Phys. Med. Rehabil..

[117] Batista E.D., Andretta A., de Miranda R.C., Nehring J., Dos Santos Paiva E., Schieferdecker M.E. (2016). Food intake assessment and quality of life in women with fibromyalgia. Rev. Bras. Reumatol. Engl. Ed..

[118] Rokyta R., Holecek V., Pekarkova I., Krejcova J., Racek J., Trefil L., Yamamotova A. (2003). Free radicals after painful stimulation are influenced by antioxidants and analgesics. Neuro Endocrinol. Lett..

[119] Mogil J.S. (2012). Pain genetics: Past, present and future. Trends Genet..

[120] Oertel B., Lotsch J. (2008). Genetic mutations that prevent pain: Implications for future pain medication. Pharmacogenomics.

[121] Arnold L.M., Fan J., Russell I.J., Yunus M.B., Khan M.A., Kushner I., Olson J.M., Iyengar S.K. (2013). The fibromyalgia family study: A genome-wide linkage scan study. Arthritis Rheum..

[122] Mergener M., Becker R.M., dos Santos A.F., dos Santos G.A., de Andrade F.M. (2011). Influence of the interaction between environmental quality and T102C SNP in the HTR2A gene on fibromyalgia susceptibility. Rev. Bras. Reum..

[123] Mickle A.D., Shepherd A.J., Mohapatra D.P. (2015). Sensory TRP channels: The key transducers of nociception and pain. Prog. Mol. Biol. Transl. Sci..

[124] Lee Y.H., Choi S.J., Ji J.D., Song G.G. (2012). Candidate gene studies of fibromyalgia: A systematic review and meta-analysis. Rheumatol. Int..

[125] Docampo E., Escaramis G., Gratacos M., Villatoro S., Puig A., Kogevinas M., Collado A., Carbonell J., Rivera J., Vidal J. (2014). Genome-wide analysis of single nucleotide polymorphisms and copy number variants in fibromyalgia suggest a role for the central nervous system. Pain.

[126] Wood P.B., Schweinhardt P., Jaeger E., Dagher A., Hakyemez H., Rabiner E.A., Bushnell M.C., Chizh B.A. (2007). Fibromyalgia patients show an abnormal dopamine response to pain. Eur. J. Neurosci..

[127] Gold S.J., Ni Y.G., Dohlman H.G., Nestler E.J. (1997). Regulators of G-protein signaling (RGS) proteins: Region-specific expression of nine subtypes in rat brain. J. Neurosci..

[128] Lu A.T., Ogdie M.N., Jarvelin M.R., Moilanen I.K., Loo S.K., McCracken J.T., McGough J.J., Yang M.H., Peltonen L., Nelson S.F. (2008). Association of the cannabinoid receptor gene (CNR1) with ADHD and post-traumatic stress disorder. Am. J. Med. Genet. B Neuropsychiatr. Genet..

[129] Park J.M., Choi M.G., Cho Y.K., Lee I.S., Kim S.W., Choi K.Y., Chung I.S. (2011). Cannabinoid receptor 1 gene polymorphism and irritable bowel syndrome in the Korean population: A hypothesis-generating study. J. Clin. Gastroenterol..

[130] Bleakman D., Alt A., Nisenbaum E.S. (2006). Glutamate receptors and pain. Semin. Cell Dev. Biol..

[131] Crofford L.J., Pillemer S.R., Kalogeras K.T., Cash J.M., Michelson D., Kling M.A., Sternberg E.M., Gold P.W., Chrousos G.P., Wilder R.L. (1994). Hypothalamic-pituitary-adrenal axis perturbations in patients with fibromyalgia. Arthritis Rheum..

[132] McCain G.A., Tilbe K.S. (1989). Diurnal hormone variation in fibromyalgia syndrome: A comparison with rheumatoid arthritis. J. Rheumatol. Suppl..

[133] McLean S.A., Williams D.A., Harris R.E., Kop W.J., Groner K.H., Ambrose K., Lyden A.K., Gracely R.H., Crofford L.J., Geisser M.E. (2005). Momentary relationship between cortisol secretion and symptoms in patients with fibromyalgia. Arthritis Rheum..

[134] Weissbecker I., Floyd A., Dedert E., Salmon P., Sephton S. (2006). Childhood trauma and diurnal cortisol disruption in fibromyalgia syndrome. Psychoneuroendocrinology.

[135] Crofford L.J., Young E.A., Engleberg N.C., Korszun A., Brucksch C.B., McClure L.A., Brown M.B., Demitrack M.A. (2004). Basal circadian and pulsatile ACTH and cortisol secretion in patients with fibromyalgia and/or chronic fatigue syndrome. Brain Behav. Immun..

[136] Abeles A.M., Pillinger M.H., Solitar B.M., Abeles M. (2007). Narrative review: The pathophysiology of fibromyalgia. Ann. Intern. Med..

[137] Rea K., Dinan T.G., Cryan J.F. (2016). The microbiome: A key regulator of stress and neuroinflammation. Neurobiol. Stress.

[138] McLean S.A., Williams D.A., Stein P.K., Harris R.E., Lyden A.K., Whalen G., Park K.M., Liberzon I., Sen A., Gracely R.H. (2006). Cerebrospinal fluid corticotropin-releasing factor concentration is associated with pain but not fatigue symptoms in patients with fibromyalgia. Neuropsychopharmacology.

[139] Wingenfeld K., Heim C., Schmidt I., Wagner D., Meinlschmidt G., Hellhammer D.H. (2008). HPA axis reactivity and lymphocyte glucocorticoid sensitivity in fibromyalgia syndrome and chronic pelvic pain. Psychosom. Med..

[140] Bennett R.M., Cook D.M., Clark S.R., Burckhardt C.S., Campbell S.M. (1997). Hypothalamic-pituitary-insulin-like growth factor-I axis dysfunction in patients with fibromyalgia. J. Rheumatol..

[141] Paul-Savoie E., Marchand S., Morin M., Bourgault P., Brissette N., Rattanavong V., Cloutier C., Bissonnette A., Potvin S. (2012). Is the deficit in pain inhibition in fibromyalgia influenced by sleep impairments?. Open Rheumatol. J..

[142] Okifuji A., Turk D.C. (2006). Sex hormones and pain in regularly menstruating women with fibromyalgia syndrome. J. Pain.

[143] Koca T., Kocyigit B., Seyithanoglu M., Berk E. (2019). The Importance of G-protein Coupled Estrogen Receptor in Patients with Fibromyalgia. Arch. Rheumatol..

[144] Epstein S.A., Kay G., Clauw D., Heaton R., Klein D., Krupp L., Kuck J., Leslie V., Masur D., Wagner M. (1999). Psychiatric disorders in patients with fibromyalgia. A multicenter investigation. Psychosomatics.

[145] Leino P., Magni G. (1993). Depressive and distress symptoms as predictors of low back pain, neck-shoulder pain, and other musculoskeletal morbidity: A 10-year follow-up of metal industry employees. Pain.

[146] Giesecke T., Gracely R.H., Williams D.A., Geisser M.E., Petzke F.W., Clauw D.J. (2005). The relationship between depression, clinical pain, and experimental pain in a chronic pain cohort. Arthritis Rheum..

[147] O’Malley P.G., Balden E., Tomkins G., Santoro J., Kroenke K., Jackson J.L. (2000). Treatment of fibromyalgia with antidepressants: A meta-analysis. J. Gen. Intern. Med..

[148] Jackson J.L., O’Malley P.G., Tomkins G., Balden E., Santoro J., Kroenke K. (2000). Treatment of functional gastrointestinal disorders with antidepressant medications: A meta-analysis. Am. J. Med..

[149] Tomkins G.E., Jackson J.L., O’Malley P.G., Balden E., Santoro J.E. (2001). Treatment of chronic headache with antidepressants: A meta-analysis. Am. J. Med..

[150] Moret C., Briley M. (2006). Antidepressants in the treatment of fibromyalgia. Neuropsychiatr. Dis. Treat..

[151] Jennings E.M., Okine B.N., Roche M., Finn D.P. (2014). Stress-induced hyperalgesia. Prog. Neurobiol..

[152] Michaux G.P.N., Magerl W., Anton F., Treede R.D. (2012). Experimental characterization of the effects of acute stresslike doses of hydrocortisone in human neurogenic hyperalgesia models. Pain.

[153] Kuehl L.K., Michaux G.P., Richter S., Schachinger H., Anton F. (2010). Increased basal mechanical pain sensitivity but decreased perceptual wind-up in a human model of relative hypocortisolism. Pain.

[154] Suarez-Roca H., Silva J.A., Arcaya J.L., Quintero L., Maixner W., Pinerua-Shuhaibar L. (2006). Role of mu-opioid and NMDA receptors in the development and maintenance of repeated swim stress-induced thermal hyperalgesia. Behav. Brain Res..

[155] Nasu T., Taguchi T., Mizumura K. (2010). Persistent deep mechanical hyperalgesia induced by repeated cold stress in rats. Eur. J. Pain.

[156] Nishiyori M., Uchida H., Nagai J., Araki K., Mukae T., Kishioka S., Ueda H. (2011). Permanent relief from intermittent cold stress-induced fibromyalgia-like abnormal pain by repeated intrathecal administration of antidepressants. Mol. Pain.

[157] Khasar S.G., Dina O.A., Green P.G., Levine J.D. (2009). Sound stress-induced long-term enhancement of mechanical hyperalgesia in rats is maintained by sympathoadrenal catecholamines. J. Pain.

[158] Sluka K.A., Danielson J., Rasmussen L., DaSilva L.F. (2012). Exercise-induced pain requires NMDA receptor activation in the medullary raphe nuclei. Med. Sci. Sports Exerc..

[159] Quintero J.E., Dooley D.J., Pomerleau F., Huettl P., Gerhardt G.A. (2011). Amperometric measurement of glutamate release modulation by gabapentin and pregabalin in rat neocortical slices: Role of voltage-sensitive Ca2+ alpha2delta-1 subunit. J. Pharm. Exp..

[160] Suarez-Roca H., Leal L., Silva J.A., Pinerua-Shuhaibar L., Quintero L. (2008). Reduced GABA neurotransmission underlies hyperalgesia induced by repeated forced swimming stress. Behav. Brain Res..

[161] Hata T., Itoh E., Kawabata A. (1991). Changes in CNS levels of serotonin and its metabolite in SART-stressed (repeatedly cold-stressed) rats. Jpn. J. Pharm..

[162] Finan P.H., Goodin B.R., Smith M.T. (2013). The association of sleep and pain: An update and a path forward. J. Pain.

[163] Mundal I., Grawe R.W., Bjorngaard J.H., Linaker O.M., Fors E.A. (2014). Prevalence and long-term predictors of persistent chronic widespread pain in the general population in an 11-year prospective study: The HUNT study. BMC Musculoskelet. Disord..

[164] Haack M., Sanchez E., Mullington J.M. (2007). Elevated inflammatory markers in response to prolonged sleep restriction are associated with increased pain experience in healthy volunteers. Sleep.

[165] Irwin M.R., Olmstead R., Carrillo C., Sadeghi N., Fitzgerald J.D., Ranganath V.K., Nicassio P.M. (2012). Sleep loss exacerbates fatigue, depression, and pain in rheumatoid arthritis. Sleep.

[166] Smith M.T., Edwards R.R., McCann U.D., Haythornthwaite J.A. (2007). The effects of sleep deprivation on pain inhibition and spontaneous pain in women. Sleep.

[167] Parrino L., Grassi A., Milioli G. (2014). Cyclic alternating pattern in polysomnography: What is it and what does it mean?. Curr. Opin. Pulm. Med..

[168] Rizzi M., Sarzi-Puttini P., Atzeni F., Capsoni F., Andreoli A., Pecis M., Colombo S., Carrabba M., Sergi M. (2004). Cyclic alternating pattern: A new marker of sleep alteration in patients with fibromyalgia?. J. Rheumatol..

[169] Tiede W., Magerl W., Baumgartner U., Durrer B., Ehlert U., Treede R.D. (2010). Sleep restriction attenuates amplitudes and attentional modulation of pain-related evoked potentials, but augments pain ratings in healthy volunteers. Pain.

[170] Moldofsky H., Scarisbrick P., England R., Smythe H. (1975). Musculosketal symptoms and non-REM sleep disturbance in patients with “fibrositis syndrome” and healthy subjects. Psychosom. Med..

[171] Gottshall J.L., Adams Z.M., Forgacs P.B., Schiff N.D. (2019). Daytime Central Thalamic Deep Brain Stimulation Modulates Sleep Dynamics in the Severely Injured Brain: Mechanistic Insights and a Novel Framework for Alpha-Delta Sleep Generation. Front. Neurol..

[172] Vijayan S., Klerman E.B., Adler G.K., Kopell N.J. (2015). Thalamic mechanisms underlying alpha-delta sleep with implications for fibromyalgia. J. Neurophysiol..

[173] Roizenblatt S., Moldofsky H., Benedito-Silva A.A., Tufik S. (2001). Alpha sleep characteristics in fibromyalgia. Arthritis Rheum..

[174] Steriade M. (2000). Corticothalamic resonance, states of vigilance and mentation. Neuroscience.

[175] Steriade M., Deschenes M. (1984). The thalamus as a neuronal oscillator. Brain Res..

[176] Steriade M., McCormick D.A., Sejnowski T.J. (1993). Thalamocortical oscillations in the sleeping and aroused brain. Science.

[177] Steriade M., Amzica F. (1998). Coalescence of sleep rhythms and their chronology in corticothalamic networks. Sleep Res. Online.

[178] Principe J.C., Smith J.R. (1982). Sleep spindle characteristics as a function of age. Sleep.

[179] Landis C.A., Lentz M.J., Rothermel J., Buchwald D., Shaver J.L. (2004). Decreased sleep spindles and spindle activity in midlife women with fibromyalgia and pain. Sleep.

[180] Kulkarni P.M., Xiao Z., Robinson E.J., Jami A.S., Zhang J., Zhou H., Henin S.E., Liu A.A., Osorio R.S., Wang J. (2019). A deep learning approach for real-time detection of sleep spindles. J. Neural Eng..

[181] Caravan B., Hu L., Veyg D., Kulkarni P., Zhang Q., Chen Z.S., Wang J. (2020). Sleep spindles as a diagnostic and therapeutic target for chronic pain. Mol. Pain.

[182] Granovsky Y., Matre D., Sokolik A., Lorenz J., Casey K.L. (2005). Thermoreceptive innervation of human glabrous and hairy skin: A contact heat evoked potential analysis. Pain.

[183] Grayston R., Czanner G., Elhadd K., Goebel A., Frank B., Uceyler N., Malik R.A., Alam U. (2019). A systematic review and meta-analysis of the prevalence of small fiber pathology in fibromyalgia: Implications for a new paradigm in fibromyalgia etiopathogenesis. Semin. Arthritis Rheum..

[184] Uceyler N., Sommer C. (2015). Fibromyalgia syndrome: A disease of the small nerve fibers?. Z. Rheumatol..

[185] Yam M.F., Loh Y.C., Tan C.S., Khadijah Adam S., Abdul Manan N., Basir R. (2018). General Pathways of Pain Sensation and the Major Neurotransmitters Involved in Pain Regulation. Int. J. Mol. Sci..

[186] Chiu I.M., von Hehn C.A., Woolf C.J. (2012). Neurogenic inflammation and the peripheral nervous system in host defense and immunopathology. Nat. Neurosci..

[187] Sorkin L.S., Eddinger K.A., Woller S.A., Yaksh T.L. (2018). Origins of antidromic activity in sensory afferent fibers and neurogenic inflammation. Semin. Immunopathol..

[188] Lerma C., Martinez A., Ruiz N., Vargas A., Infante O., Martinez-Lavin M. (2011). Nocturnal heart rate variability parameters as potential fibromyalgia biomarker: Correlation with symptoms severity. Arthritis Res..

[189] Lerma C., Martinez-Martinez L.A., Ruiz N., Vargas A., Infante O., Martinez-Lavin M. (2016). Fibromyalgia beyond reductionism. Heart rhythm fractal analysis to assess autonomic nervous system resilience. Scand. J. Rheumatol..

[190] Dawson L.F., Phillips J.K., Finch P.M., Inglis J.J., Drummond P.D. (2011). Expression of alpha1-adrenoceptors on peripheral nociceptive neurons. Neuroscience.

[191] Maestroni G.J. (2006). Sympathetic nervous system influence on the innate immune response. Ann. N. Y. Acad. Sci..

[192] Kim S.H., Kim D.H., Oh D.H., Clauw D.J. (2008). Characteristic electron microscopic findings in the skin of patients with fibromyalgia--preliminary study. Clin. Rheumatol..

[193] Leinders M., Doppler K., Klein T., Deckart M., Rittner H., Sommer C., Uceyler N. (2016). Increased cutaneous miR-let-7d expression correlates with small nerve fiber pathology in patients with fibromyalgia syndrome. Pain.

[194] Harte S.E., Clauw D.J., Hayes J.M., Feldman E.L., St Charles I.C., Watson C.J. (2017). Reduced intraepidermal nerve fiber density after a sustained increase in insular glutamate: A proof-of-concept study examining the pathogenesis of small fiber pathology in fibromyalgia. Pain Rep..

[195] McLean S.A., Clauw D.J. (2005). Biomedical models of fibromyalgia. Disabil. Rehabil..

[196] Schrepf A., Moser S., Harte S.E., Basu N., Kaplan C., Kolarik E., Tsodikov A., Brummett C.M., Clauw D.J. (2020). Top down or bottom up? An observational investigation of improvement in fibromyalgia symptoms following hip and knee replacement. Rheumatology.

[197] Baron R., Hans G., Dickenson A.H. (2013). Peripheral input and its importance for central sensitization. Ann. Neurol..

[198] Wolfe F., Smythe H.A., Yunus M.B., Bennett R.M., Bombardier C., Goldenberg D.L., Tugwell P., Campbell S.M., Abeles M., Clark P. (1990). The American College of Rheumatology 1990 Criteria for the Classification of Fibromyalgia. Report of the Multicenter Criteria Committee. Arthritis Rheum..

[199] Julien N., Goffaux P., Arsenault P., Marchand S. (2005). Widespread pain in fibromyalgia is related to a deficit of endogenous pain inhibition. Pain.

[200] Sluka K.A., Clauw D.J. (2016). Neurobiology of fibromyalgia and chronic widespread pain. Neuroscience.

[201] Ablin J.N., Buskila D. (2015). Update on the genetics of the fibromyalgia syndrome. Best Pract. Res. Clin. Rheumatol..

[202] D’Agnelli S., Arendt-Nielsen L., Gerra M.C., Zatorri K., Boggiani L., Baciarello M., Bignami E. (2019). Fibromyalgia: Genetics and epigenetics insights may provide the basis for the development of diagnostic biomarkers. Mol. Pain.

[203] Tour J., Lofgren M., Mannerkorpi K., Gerdle B., Larsson A., Palstam A., Bileviciute-Ljungar I., Bjersing J., Martin I., Ernberg M. (2017). Gene-to-gene interactions regulate endogenous pain modulation in fibromyalgia patients and healthy controls-antagonistic effects between opioid and serotonin-related genes. Pain.

[204] Offenbaecher M., Bondy B., de Jonge S., Glatzeder K., Kruger M., Schoeps P., Ackenheil M. (1999). Possible association of fibromyalgia with a polymorphism in the serotonin transporter gene regulatory region. Arthritis Rheum..

[205] Cohen H., Buskila D., Neumann L., Ebstein R.P. (2002). Confirmation of an association between fibromyalgia and serotonin transporter promoter region (5- HTTLPR) polymorphism, and relationship to anxiety-related personality traits. Arthritis Rheum..

[206] Gursoy S. (2002). Absence of association of the serotonin transporter gene polymorphism with the mentally healthy subset of fibromyalgia patients. Clin. Rheumatol..

[207] Martinez-Jauand M., Sitges C., Rodriguez V., Picornell A., Ramon M., Buskila D., Montoya P. (2013). Pain sensitivity in fibromyalgia is associated with catechol-O-methyltransferase (COMT) gene. Eur. J. Pain.

[208] Inanir A., Karakus N., Ates O., Sezer S., Bozkurt N., Inanir S., Yigit S. (2014). Clinical symptoms in fibromyalgia are associated to catechol-O-methyltransferase (COMT) gene Val158Met polymorphism. Xenobiotica.

[209] Cohen H., Neumann L., Glazer Y., Ebstein R.P., Buskila D. (2009). The relationship between a common catechol-O-methyltransferase (COMT) polymorphism val(158) met and fibromyalgia. Clin. Exp. Rheumatol..

[210] Lee Y.H., Kim J.H., Song G.G. (2015). Association between the COMT Val158Met polymorphism and fibromyalgia susceptibility and fibromyalgia impact questionnaire score: A meta-analysis. Rheumatol. Int..

[211] Smith S.B., Maixner D.W., Fillingim R.B., Slade G., Gracely R.H., Ambrose K., Zaykin D.V., Hyde C., John S., Tan K. (2012). Large candidate gene association study reveals genetic risk factors and therapeutic targets for fibromyalgia. Arthritis Rheum..

[212] Solak O., Erdogan M.O., Yildiz H., Ulasli A.M., Yaman F., Terzi E.S., Ulu S., Dundar U., Solak M. (2014). Assessment of opioid receptor mu1 gene A118G polymorphism and its association with pain intensity in patients with fibromyalgia. Rheumatol. Int..

[213] Arnold L.M., Bennett R.M., Crofford L.J., Dean L.E., Clauw D.J., Goldenberg D.L., Fitzcharles M.A., Paiva E.S., Staud R., Sarzi-Puttini P. (2019). AAPT Diagnostic Criteria for Fibromyalgia. J. Pain.

[214] Park D.J., Lee S.S. (2017). New insights into the genetics of fibromyalgia. Korean J. Intern. Med..

[215] Garcia Rodriguez D.F., Abud Mendoza C. (2020). Physiopathology of fibromyalgia. Reum. Clin..

[216] Dolcino M., Tinazzi E., Puccetti A., Lunardi C. (2020). Gene Expression Profiling in Fibromyalgia Indicates an Autoimmune Origin of the Disease and Opens New Avenues for Targeted Therapy. J. Clin. Med..

[217] Park D.J., Kim S.H., Nah S.S., Lee J.H., Kim S.K., Lee Y.A., Hong S.J., Kim H.S., Lee H.S., Kim H.A. (2016). Polymorphisms of the TRPV2 and TRPV3 genes associated with fibromyalgia in a Korean population. Rheumatology.

[218] Caccamo D., Cesareo E., Mariani S., Raskovic D., Ientile R., Curro M., Korkina L., De Luca C. (2013). Xenobiotic sensor- and metabolism-related gene variants in environmental sensitivity-related illnesses: A survey on the Italian population. Oxid. Med. Cell Longev..

[219] Xiao Y., Russell I.J., Liu Y.G. (2012). A brain-derived neurotrophic factor polymorphism Val66Met identifies fibromyalgia syndrome subgroup with higher body mass index and C-reactive protein. Rheumatol. Int..

[220] Haas L., Portela L.V., Bohmer A.E., Oses J.P., Lara D.R. (2010). Increased plasma levels of brain derived neurotrophic factor (BDNF) in patients with fibromyalgia. Neurochem. Res..

[221] Park D.J., Kim S.H., Nah S.S., Lee J.H., Kim S.K., Lee Y.A., Hong S.J., Kim H.S., Lee H.S., Kim H.A. (2018). Association between brain-derived neurotrophic factor gene polymorphisms and fibromyalgia in a Korean population: A multicenter study. Arthritis Res..

[222] Nugraha B., Anwar S.L., Gutenbrunner C., Korallus C. (2020). Polymorphisms of brain-derived neurotrophic factor genes are associated with anxiety and body mass index in fibromyalgia syndrome patients. BMC Res. Notes.

[223] Polli A., Ghosh M., Bakusic J., Ickmans K., Monteyne D., Velkeniers B., Bekaert B., Godderis L., Nijs J. (2020). DNA Methylation and Brain-Derived Neurotrophic Factor Expression Account for Symptoms and Widespread Hyperalgesia in Patients with Chronic Fatigue Syndrome and Comorbid Fibromyalgia. Arthritis Rheumatol..

[224] Menzies V., Lyon D.E., Archer K.J., Zhou Q., Brumelle J., Jones K.H., Gao G., York T.P., Jackson-Cook C. (2013). Epigenetic alterations and an increased frequency of micronuclei in women with fibromyalgia. Nurs. Res. Pract..

[225] Burri A., Marinova Z., Robinson M.D., Kuhnel B., Waldenberger M., Wahl S., Kunze S., Gieger C., Livshits G., Williams F. (2016). Are Epigenetic Factors Implicated in Chronic Widespread Pain?. PLoS ONE.

[226] Jones K.D., Gelbart T., Whisenant T.C., Waalen J., Mondala T.S., Ikle D.N., Salomon D.R., Bennett R.M., Kurian S.M. (2016). Genome-wide expression profiling in the peripheral blood of patients with fibromyalgia. Clin. Exp. Rheumatol..

[227] Buskila D., Cohen H., Neumann L., Ebstein R.P. (2004). An association between fibromyalgia and the dopamine D4 receptor exon III repeat polymorphism and relationship to novelty seeking personality traits. Mol. Psychiatry.

[228] Gursoy S., Erdal E., Herken H., Madenci E., Alasehirli B., Erdal N. (2003). Significance of catechol-O-methyltransferase gene polymorphism in fibromyalgia syndrome. Rheumatol. Int..

[229] Finan P.H., Zautra A.J., Davis M.C., Lemery-Chalfant K., Covault J., Tennen H. (2010). Genetic influences on the dynamics of pain and affect in fibromyalgia. Health Psychol..

[230] Vargas-Alarcon G., Fragoso J.M., Cruz-Robles D., Vargas A., Martinez A., Lao-Villadoniga J.I., Garcia-Fructuoso F., Vallejo M., Martinez-Lavin M. (2009). Association of adrenergic receptor gene polymorphisms with different fibromyalgia syndrome domains. Arthritis Rheum..

[231] Szyf M., Bick J. (2013). DNA methylation: A mechanism for embedding early life experiences in the genome. Child. Dev..

[232] Denk F., McMahon S.B. (2012). Chronic pain: Emerging evidence for the involvement of epigenetics. Neuron.

[233] Wang F., Stefano G.B., Kream R.M. (2014). Epigenetic modification of DRG neuronal gene expression subsequent to nerve injury: Etiological contribution to complex regional pain syndromes (Part II). Med. Sci. Monit..

[234] Seo S., Grzenda A., Lomberk G., Ou X.M., Cruciani R.A., Urrutia R. (2013). Epigenetics: A promising paradigm for better understanding and managing pain. J. Pain.

[235] Lewis B.P., Burge C.B., Bartel D.P. (2005). Conserved seed pairing, often flanked by adenosines, indicates that thousands of human genes are microRNA targets. Cell.

[236] Baek D., Villen J., Shin C., Camargo F.D., Gygi S.P., Bartel D.P. (2008). The impact of microRNAs on protein output. Nature.

[237] Andersen H.H., Duroux M., Gazerani P. (2014). MicroRNAs as modulators and biomarkers of inflammatory and neuropathic pain conditions. Neurobiol. Dis..

[238] Bartel D.P. (2004). MicroRNAs: Genomics, biogenesis, mechanism, and function. Cell.

[239] Bjersing J.L., Lundborg C., Bokarewa M.I., Mannerkorpi K. (2013). Profile of cerebrospinal microRNAs in fibromyalgia. PLoS ONE.

[240] Masotti A., Baldassarre A., Guzzo M.P., Iannuccelli C., Barbato C., Di Franco M. (2017). Circulating microRNA Profiles as Liquid Biopsies for the Characterization and Diagnosis of Fibromyalgia Syndrome. Mol. Neurobiol..

[241] Nakamura T., Schwander S.K., Donnelly R., Ortega F., Togo F., Broderick G., Yamamoto Y., Cherniack N.S., Rapoport D., Natelson B.H. (2010). Cytokines across the night in chronic fatigue syndrome with and without fibromyalgia. Clin. Vaccine Immunol..

[242] Montana M.C., Gereau R.W. (2011). Metabotropic glutamate receptors as targets for analgesia: Antagonism, activation, and allosteric modulation. Curr. Pharm. Biotechnol..

[243] Raffaeli W., Malafoglia V., Bonci A., Tenti M., Ilari S., Gremigni P., Iannuccelli C., Gioia C., Di Franco M., Mollace V. (2020). Identification of MOR-Positive B Cell as Possible Innovative Biomarker (Mu Lympho-Marker) for Chronic Pain Diagnosis in Patients with Fibromyalgia and Osteoarthritis Diseases. Int. J. Mol. Sci..

[244] Applbaum E., Lichtbroun A. (2019). Novel Sjogren’s autoantibodies found in fibromyalgia patients with sicca and/or xerostomia. Autoimmun. Rev..

[245] Nishikai M., Tomomatsu S., Hankins R.W., Takagi S., Miyachi K., Kosaka S., Akiya K. (2001). Autoantibodies to a 68/48 kDa protein in chronic fatigue syndrome and primary fibromyalgia: A possible marker for hypersomnia and cognitive disorders. Rheumatology.

[246] Arora N., Gupta A., Reddy S.B. (2017). Antinuclear Antibody and Subserology Testing in the Evaluation of Fibromyalgia: A Teachable Moment. JAMA Intern. Med..

[247] Kotter I., Neuscheler D., Gunaydin I., Wernet D., Klein R. (2007). Is there a predisposition for the development of autoimmune diseases in patients with fibromyalgia? Retrospective analysis with long term follow-up. Rheumatol. Int..

[248] Jensen B., Wittrup I.H., Wiik A., Bliddal H., Friis A.S., McLaughlin J.K., Danneskiold-Samsoe B., Olsen J.H. (2004). Antipolymer antibodies in Danish fibromyalgia patients. Clin. Exp. Rheumatol..

[249] Di Franco M., Iannuccelli C., Alessandri C., Paradiso M., Riccieri V., Libri F., Valesini G. (2009). Autonomic dysfunction and neuropeptide Y in fibromyalgia. Clin. Exp. Rheumatol..

[250] Staines D.R. (2004). Is fibromyalgia an autoimmune disorder of endogenous vasoactive neuropeptides?. Med. Hypotheses.

[251] Tsilioni I., Russell I.J., Stewart J.M., Gleason R.M., Theoharides T.C. (2016). Neuropeptides CRH, SP, HK-1, and Inflammatory Cytokines IL-6 and TNF Are Increased in Serum of Patients with Fibromyalgia Syndrome, Implicating Mast Cells. J. Pharm. Exp..

[252] Laske C., Stransky E., Eschweiler G.W., Klein R., Wittorf A., Leyhe T., Richartz E., Kohler N., Bartels M., Buchkremer G. (2007). Increased BDNF serum concentration in fibromyalgia with or without depression or antidepressants. J. Psychiatr. Res..

[253] Pyke T.L., Osmotherly P.G., Baines S. (2017). Measuring Glutamate Levels in the Brains of Fibromyalgia Patients and a Potential Role for Glutamate in the Pathophysiology of Fibromyalgia Symptoms: A Systematic Review. Clin. J. Pain.

[254] Clos-Garcia M., Andres-Marin N., Fernandez-Eulate G., Abecia L., Lavin J.L., van Liempd S., Cabrera D., Royo F., Valero A., Errazquin N. (2019). Gut microbiome and serum metabolome analyses identify molecular biomarkers and altered glutamate metabolism in fibromyalgia. EBioMedicine.

[255] Ernberg M., Christidis N., Ghafouri B., Bileviciute-Ljungar I., Lofgren M., Bjersing J., Palstam A., Larsson A., Mannerkorpi K., Gerdle B. (2018). Plasma Cytokine Levels in Fibromyalgia and Their Response to 15 Weeks of Progressive Resistance Exercise or Relaxation Therapy. Mediat. Inflamm..

[256] Wallace D.J., Linker-Israeli M., Hallegua D., Silverman S., Silver D., Weisman M.H. (2001). Cytokines play an aetiopathogenetic role in fibromyalgia: A hypothesis and pilot study. Rheumatology.

[257] Han C.L., Sheng Y.C., Wang S.Y., Chen Y.H., Kang J.H. (2020). Serum proteome profiles revealed dysregulated proteins and mechanisms associated with fibromyalgia syndrome in women. Sci. Rep..

[258] Menzies V., Starkweather A., Yao Y., Thacker L.R., Garrett T.J., Swift-Scanlan T., Kelly D.L., Patel P., Lyon D.E. (2020). Metabolomic Differentials in Women with and Without Fibromyalgia. Clin. Transl. Sci..

[259] Menzies V., Starkweather A., Yao Y., Kelly D.L., Garrett T.J., Yang G., Booker S., Swift-Scanlan T., Mahmud I., Lyon D.E. (2021). Exploring Associations Between Metabolites and Symptoms of Fatigue, Depression and Pain in Women with Fibromyalgia. Biol. Res. Nurs..

[260] Caboni P., Liori B., Kumar A., Santoru M.L., Asthana S., Pieroni E., Fais A., Era B., Cacace E., Ruggiero V. (2014). Metabolomics analysis and modeling suggest a lysophosphocholines-PAF receptor interaction in fibromyalgia. PLoS ONE.

[261] Malatji B.G., Meyer H., Mason S., Engelke U.F.H., Wevers R.A., van Reenen M., Reinecke C.J. (2017). A diagnostic biomarker profile for fibromyalgia syndrome based on an NMR metabolomics study of selected patients and controls. BMC Neurol..

[262] Hackshaw K.V., Rodriguez-Saona L., Plans M., Bell L.N., Buffington C.A. (2013). A bloodspot-based diagnostic test for fibromyalgia syndrome and related disorders. Analyst.

[263] Hackshaw K.V., Aykas D.P., Sigurdson G.T., Plans M., Madiai F., Yu L., Buffington C.A.T., Giusti M.M., Rodriguez-Saona L. (2019). Metabolic fingerprinting for diagnosis of fibromyalgia and other rheumatologic disorders. J. Biol. Chem..

[264] Wolfe F. (1999). “Silicone related symptoms” are common in patients with fibromyalgia: No evidence for a new disease. J. Rheumatol..

[265] Iannuccelli C., Di Franco M., Alessandri C., Guzzo M.P., Croia C., Di Sabato F., Foti M., Valesini G. (2010). Pathophysiology of fibromyalgia: A comparison with the tension-type headache, a localized pain syndrome. Ann. N. Y. Acad. Sci.

[266] Klein R., Berg P.A. (1995). High incidence of antibodies to 5-hydroxytryptamine, gangliosides and phospholipids in patients with chronic fatigue and fibromyalgia syndrome and their relatives: Evidence for a clinical entity of both disorders. Eur. J. Med. Res..

[267] Werle E., Fischer H.P., Muller A., Fiehn W., Eich W. (2001). Antibodies against serotonin have no diagnostic relevance in patients with fibromyalgia syndrome. J. Rheumatol..

[268] Bazzichi L., Rossi A., Giacomelli C., Bombardieri S. (2010). Exploring the abyss of fibromyalgia biomarkers. Clin. Exp. Rheumatol..

[269] Pamuk O.N., Cakir N. (2007). The frequency of thyroid antibodies in fibromyalgia patients and their relationship with symptoms. Clin. Rheumatol..

[270] Bazzichi L., Rossi A., Zirafa C., Monzani F., Tognini S., Dardano A., Santini F., Tonacchera M., De Servi M., Giacomelli C. (2012). Thyroid autoimmunity may represent a predisposition for the development of fibromyalgia?. Rheumatol. Int..

[271] Ciregia F., Giacomelli C., Giusti L., Bazzichi L., Lucacchini A., Eilke W.S. (2012). Diagnosis of Fibromyalgia Syndrome:Potential Biomarkers and Proteomic Approach. New Insights into Fibromyalgia.

[272] Maes M., Verkerk R., Delmeire L., Van Gastel A., van Hunsel F., Scharpe S. (2000). Serotonergic markers and lowered plasma branched-chain-amino acid concentrations in fibromyalgia. Psychiatry Res..

[273] Bazzichi L., Palego L., Giannaccini G., Rossi A., De Feo F., Giacomelli C., Betti L., Giusti L., Mascia G., Bombardieri S. (2009). Altered amino acid homeostasis in subjects affected by fibromyalgia. Clin. Biochem..

[274] Anderberg U.M., Liu Z., Berglund L., Nyberg F. (1999). Elevated plasma levels of neuropeptide Y in female fibromyalgia patients. Eur. J. Pain.

[275] Evengard B., Nilsson C.G., Lindh G., Lindquist L., Eneroth P., Fredrikson S., Terenius L., Henriksson K.G. (1998). Chronic fatigue syndrome differs from fibromyalgia. No evidence for elevated substance P levels in cerebrospinal fluid of patients with chronic fatigue syndrome. Pain.

[276] Andersen M.L., Nascimento D.C., Machado R.B., Roizenblatt S., Moldofsky H., Tufik S. (2006). Sleep disturbance induced by substance P in mice. Behav. Brain Res..

[277] Sarchielli P., Mancini M.L., Floridi A., Coppola F., Rossi C., Nardi K., Acciarresi M., Pini L.A., Calabresi P. (2007). Increased levels of neurotrophins are not specific for chronic migraine: Evidence from primary fibromyalgia syndrome. J. Pain.

[278] Valdes M., Collado A., Bargallo N., Vazquez M., Rami L., Gomez E., Salamero M. (2010). Increased glutamate/glutamine compounds in the brains of patients with fibromyalgia: A magnetic resonance spectroscopy study. Arthritis Rheum..

[279] Bazzichi L., Ciregia F., Giusti L., Baldini C., Giannaccini G., Giacomelli C., Sernissi F., Bombardieri S., Lucacchini A. (2009). Detection of potential markers of primary fibromyalgia syndrome in human saliva. Proteom. Clin. Appl..

[280] Bagis S., Tamer L., Sahin G., Bilgin R., Guler H., Ercan B., Erdogan C. (2005). Free radicals and antioxidants in primary fibromyalgia: An oxidative stress disorder?. Rheumatol. Int..

[281] Miranda-Díaz A.G., Rodríguez-Lara S.Q., Wilke W.S. (2018). The Role of Oxidants/Antioxidants, Mitochondrial. Dysfunction, and Autophagy in Fibromyalgia. Discussions of Unusual Topics in Fibromyalgia.

[282] Quintans-Junior L.J., Brito R.G., Quintans J.S.S., Santos P.L., Camargo Z.T., Barreto P.A., Arrigoni-Blank M.F., Lucca-Junior W., Scotti L., Scotti M.T. (2018). Nanoemulsion Thermoreversible Pluronic F127-Based Hydrogel Containing Hyptis pectinata (Lamiaceae) Leaf Essential Oil Produced a Lasting Anti-hyperalgesic Effect in Chronic Noninflammatory Widespread Pain in Mice. Mol. Neurobiol..

[283] Lister R.E. (2002). An open, pilot study to evaluate the potential benefits of coenzyme Q10 combined with Ginkgo biloba extract in fibromyalgia syndrome. J. Int. Med. Res..

[284] Oliveira M.G., Brito R.G., Santos P.L., Araujo-Filho H.G., Quintans J.S., Menezes P.P., Serafini M.R., Carvalho Y.M., Silva J.C., Almeida J.R. (2016). Alpha-Terpineol, a monoterpene alcohol, complexed with beta-cyclodextrin exerts antihyperalgesic effect in animal model for fibromyalgia aided with docking study. Chem. Biol. Interact..

[285] Quintans-Junior L.J., Araujo A.A., Brito R.G., Santos P.L., Quintans J.S., Menezes P.P., Serafini M.R., Silva G.F., Carvalho F.M., Brogden N.K. (2016). Beta-caryophyllene, a dietary cannabinoid, complexed with beta-cyclodextrin produced anti-hyperalgesic effect involving the inhibition of Fos expression in superficial dorsal horn. Life Sci..

[286] Casanueva B., Rodero B., Quintial C., Llorca J., Gonzalez-Gay M.A. (2013). Short-term efficacy of topical capsaicin therapy in severely affected fibromyalgia patients. Rheumatol. Int..

[287] Fusco R., Siracusa R., D’Amico R., Peritore A.F., Cordaro M., Gugliandolo E., Crupi R., Impellizzeri D., Cuzzocrea S., Di Paola R. (2019). Melatonin Plus Folic Acid Treatment Ameliorates Reserpine-Induced Fibromyalgia: An Evaluation of Pain, Oxidative Stress, and Inflammation. Antioxidants.

[288] de Zanette S.A., Vercelino R., Laste G., Rozisky J.R., Schwertner A., Machado C.B., Xavier F., de Souza I.C., Deitos A., Torres I.L. (2014). Melatonin analgesia is associated with improvement of the descending endogenous pain-modulating system in fibromyalgia: A phase II, randomized, double-dummy, controlled trial. BMC Pharm. Toxicol..

[289] Hussain S.A., Al K., Jasim N.A., Gorial F.I. (2011). Adjuvant use of melatonin for treatment of fibromyalgia. J. Pineal Res..

[290] Cordero M.D., Cotan D., del-Pozo-Martin Y., Carrion A.M., de Miguel M., Bullon P., Sanchez-Alcazar J.A. (2012). Oral coenzyme Q10 supplementation improves clinical symptoms and recovers pathologic alterations in blood mononuclear cells in a fibromyalgia patient. Nutrition.

[291] Cordero M.D., Cano-Garcia F.J., Alcocer-Gomez E., De Miguel M., Sanchez-Alcazar J.A. (2012). Oxidative stress correlates with headache symptoms in fibromyalgia: Coenzyme Q(1)(0) effect on clinical improvement. PLoS ONE.

[292] Sawaddiruk P., Apaijai N., Paiboonworachat S., Kaewchur T., Kasitanon N., Jaiwongkam T., Kerdphoo S., Chattipakorn N., Chattipakorn S.C. (2019). Coenzyme Q10 supplementation alleviates pain in pregabalin-treated fibromyalgia patients via reducing brain activity and mitochondrial dysfunction. Free Radic. Res..

[293] Joustra M.L., Minovic I., Janssens K.A.M., Bakker S.J.L., Rosmalen J.G.M. (2017). Vitamin and mineral status in chronic fatigue syndrome and fibromyalgia syndrome: A systematic review and meta-analysis. PLoS ONE.

[294] Ellis S.D., Kelly S.T., Shurlock J.H., Hepburn A.L.N. (2018). The role of vitamin D testing and replacement in fibromyalgia: A systematic literature review. BMC Rheumatol..

[295] Gugliandolo E., Peritore A.F., Piras C., Cuzzocrea S., Crupi R. (2020). Palmitoylethanolamide and Related ALIAmides: Prohomeostatic Lipid Compounds for Animal Health and Wellbeing. Vet. Sci..

[296] Peritore A.F., Siracusa R., Crupi R., Cuzzocrea S. (2019). Therapeutic Efficacy of Palmitoylethanolamide and Its New Formulations in Synergy with Different Antioxidant Molecules Present in Diets. Nutrients.

[297] D’Amico R., Impellizzeri D., Cuzzocrea S., Di Paola R. (2020). ALIAmides Update: Palmitoylethanolamide and Its Formulations on Management of Peripheral Neuropathic Pain. Int. J. Mol. Sci..

[298] Impellizzeri D., Bruschetta G., Cordaro M., Crupi R., Siracusa R., Esposito E., Cuzzocrea S. (2014). Micronized/ultramicronized palmitoylethanolamide displays superior oral efficacy compared to nonmicronized palmitoylethanolamide in a rat model of inflammatory pain. J. Neuroinflam..

[299] Impellizzeri D., Peritore A.F., Cordaro M., Gugliandolo E., Siracusa R., Crupi R., D’Amico R., Fusco R., Evangelista M., Cuzzocrea S. (2019). The neuroprotective effects of micronized PEA (PEA-m) formulation on diabetic peripheral neuropathy in mice. FASEB J..

[300] Bartolucci M.L., Marini I., Bortolotti F., Impellizzeri D., Di Paola R., Bruschetta G., Crupi R., Portelli M., Militi A., Oteri G. (2018). Micronized palmitoylethanolamide reduces joint pain and glial cell activation. Inflamm. Res..

[301] Siracusa R., Fusco R., Cordaro M., Peritore A.F., D’Amico R., Gugliandolo E., Crupi R., Genovese T., Evangelista M., Di Paola R. (2020). The Protective Effects of Pre- and Post-Administration of Micronized Palmitoylethanolamide Formulation on Postoperative Pain in Rats. Int. J. Mol. Sci..

[302] Gatti A., Lazzari M., Gianfelice V., Di Paolo A., Sabato E., Sabato A.F. (2012). Palmitoylethanolamide in the treatment of chronic pain caused by different etiopathogenesis. Pain Med..

[303] Truini A., Biasiotta A., Di Stefano G., La Cesa S., Leone C., Cartoni C., Federico V., Petrucci M.T., Cruccu G. (2011). Palmitoylethanolamide restores myelinated-fibre function in patients with chemotherapy-induced painful neuropathy. CNS Neurol. Disord. Drug Targets.

[304] Schifilliti C., Cucinotta L., Fedele V., Ingegnosi C., Luca S., Leotta C. (2014). Micronized palmitoylethanolamide reduces the symptoms of neuropathic pain in diabetic patients. Pain Res. Treat..

[305] Passavanti M.B., Fiore M., Sansone P., Aurilio C., Pota V., Barbarisi M., Fierro D., Pace M.C. (2017). The beneficial use of ultramicronized palmitoylethanolamide as add-on therapy to Tapentadol in the treatment of low back pain: A pilot study comparing prospective and retrospective observational arms. BMC Anesth..

[306] Schweiger V., Martini A., Bellamoli P., Donadello K., Schievano C., Balzo G.D., Sarzi-Puttini P., Parolini M., Polati E. (2019). Ultramicronized Palmitoylethanolamide (um-PEA) as Add-on Treatment in Fibromyalgia Syndrome (FMS): Retrospective Observational Study on 407 Patients. CNS Neurol. Disord. Drug Targets.

